# EMC Is Required to Initiate Accurate Membrane Protein Topogenesis

**DOI:** 10.1016/j.cell.2018.10.009

**Published:** 2018-11-29

**Authors:** Patrick J. Chitwood, Szymon Juszkiewicz, Alina Guna, Sichen Shao, Ramanujan S. Hegde

**Affiliations:** 1 MRC Laboratory of Molecular Biology, Francis Crick Avenue, Cambridge CB2 0QH, UK; 2 Department of Cell Biology, Harvard Medical School, 240 Longwood Avenue, Boston, MA 02115, USA

## Abstract

Mammals encode ∼5,000 integral membrane proteins that need to be inserted in a defined topology at the endoplasmic reticulum (ER) membrane by mechanisms that are incompletely understood. Here, we found that efficient biogenesis of β1-adrenergic receptor (β1AR) and other G protein-coupled receptors (GPCRs) requires the conserved ER membrane protein complex (EMC). Reconstitution studies of β1AR biogenesis narrowed the EMC requirement to the co-translational insertion of the first transmembrane domain (TMD). Without EMC, a proportion of TMD1 inserted in an inverted orientation or failed altogether. Purified EMC and SRP receptor were sufficient for correctly oriented TMD1 insertion, while the Sec61 translocon was necessary for insertion of the next TMD. Enforcing TMD1 topology with an N-terminal signal peptide bypassed the EMC requirement for insertion *in vitro* and restored efficient biogenesis of multiple GPCRs in EMC-knockout cells. Thus, EMC inserts TMDs co-translationally and cooperates with the Sec61 translocon to ensure accurate topogenesis of many membrane proteins.

## Highlights

• Efficient biogenesis of many GPCRs requires EMC, the ER membrane protein complex
• Without EMC, correct topology and insertion of the first transmembrane domain fails
• Purified EMC is sufficient for insertion of the first transmembrane domain of GPCRs
• The Sec61 complex is required for insertion of subsequent transmembrane domains

## Graphical Abstract

**Figure:**
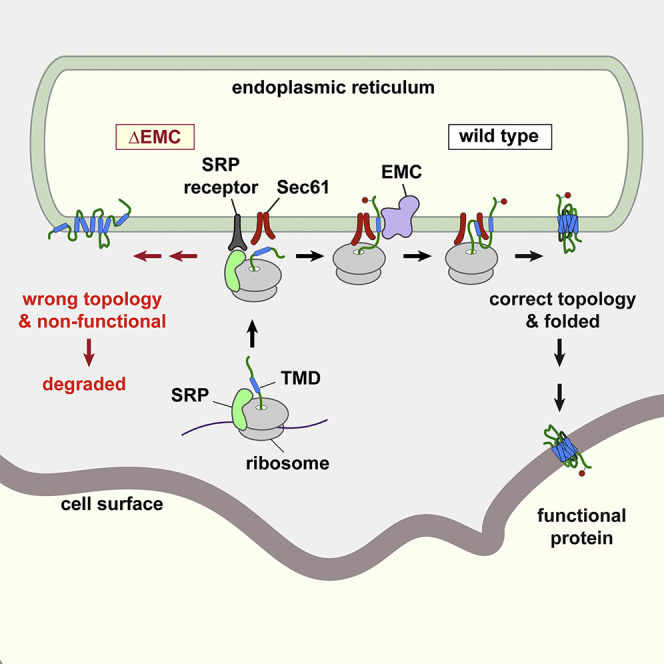

## Teaser

The ER membrane protein complex helps establish the topology of many multipass transmembrane proteins, including GPCRs, by guiding insertion of the first transmembrane domain in the correct orientation.

## Introduction

A membrane protein’s topology is determined during its initial biogenesis and is generally maintained throughout the protein’s lifetime (Shao and Hegde, 2011). The topology of a single-pass membrane protein is defined by its sole first transmembrane domain (TMD). Although multi-pass membrane proteins have more than one TMD, it is apparent from inspection of known membrane protein structures that their orientations are strongly interdependent on each other. Hence, fixing the topology of one TMD generally constrains the others, simplifying the topogenesis problem. For most multi-pass membrane proteins, the first TMD is thought to be critical for setting overall topology by essentially defining the “reading frame” for interpretation of downstream TMDs (Blobel, 1980). Thus, an understanding of membrane protein topogenesis necessarily requires knowledge of how the first TMD is recognized, oriented, and inserted into the lipid bilayer.

Of the ∼5.000 human membrane proteins inserted at the endoplasmic reticulum (ER) (UniProt Consortium, 2018), ∼64% are thought to rely on their first TMD for targeting and setting the protein’s overall topology. TMDs that mediate both targeting and insertion are termed signal anchors. The topology of a signal anchor is influenced by TMD length, its hydrophobicity, the distribution of flanking charges, and the length and folding of the preceding soluble domain (Higy et al., 2004). A folded or highly basic N-terminal domain prevents its translocation (Beltzer et al., 1991, Denzer et al., 1995), forcing the signal anchor to adopt a topology with the N terminus facing the cytosol (designated N_cyt_). Unfolded and short N-terminal domains are compatible with either topology. In this instance, N-terminal translocation to the exoplasmic side of the membrane (termed N_exo_) is favored by longer and more hydrophobic TMDs followed by positive charges (Kida et al., 2006, Wahlberg and Spiess, 1997). Despite these general trends, it has been difficult to define conclusive predictive rules (Higy et al., 2004), and many native signal anchors display ambiguous or even contradictory features.

The mechanisms by which sequence features of a signal anchor are decoded by the insertion machinery to determine topology are not clear. Reconstitution experiments showed that after targeting via the signal recognition particle (SRP) and SRP receptor (SR), the Sec61 complex is entirely sufficient for providing model signal anchors access to the lipid bilayer (Görlich and Rapoport, 1993, Heinrich et al., 2000, Oliver et al., 1995). However, analysis of various Sec61 mutations based on its structure did not provide clear explanations for how it might decode signal anchor topology (Goder et al., 2004, Junne et al., 2007). For example, extensive mutagenesis reversing the surface charges on Sec61 had surprisingly modest effects on the topology of model signal anchor sequences in yeast (Goder et al., 2004).

Recently, the highly conserved ER membrane protein complex (EMC) has been functionally and biochemically linked to membrane protein biogenesis. Since its discovery in yeast as a six-protein membrane-embedded complex needed for ER protein homeostasis (Jonikas et al., 2009), EMC has been associated with highly pleiotropic phenotypes in many organisms (Bircham et al., 2011, Lahiri et al., 2014, Louie et al., 2012, Richard et al., 2013, Satoh et al., 2015). Among them, several studies have documented reduced levels of various integral membrane proteins (Bircham et al., 2011, Richard et al., 2013, Satoh et al., 2015, Shurtleff et al., 2018), some of which are retained in the early secretory pathway. These findings suggest a broad function related to membrane protein biology, consistent with EMCs wide conservation, high abundance, residence in the ER, and widespread expression pattern (Wideman, 2015). However, the biochemical function(s) of EMC have been obscure because none of its subunits (ten in mammals) has any recognizable enzymatic activity or clear homology to proteins with well-established functions.

The only process for which a direct biochemical role of EMC has been shown is the post-translational insertion of tail-anchored membrane proteins into the ER (Guna et al., 2018). This reaction was reconstituted with purified mammalian EMC in liposomes, suggesting that EMC can directly facilitate TMD transfer from the cytosol into the lipid bilayer. Intriguingly, the EMC3 subunit shows weak resemblance to both eukaryotic Get1 and a region of prokaryotic YidC (Anghel et al., 2017). Because Get1 and YidC are both membrane protein insertases, it has been speculated that EMC might have broader roles in TMD insertion beyond tail-anchored membrane proteins (Guna and Hegde, 2018). Here, we investigated whether EMC plays a direct role in the biogenesis of G protein-coupled receptors (GPCRs), a large family of multi-pass membrane proteins of exceptional importance to nearly all aspects of human physiology.

## Results

### EMC Is Required for Optimal β_1_-Adrenergic Receptor Biogenesis in Cells

**Figure 1:**
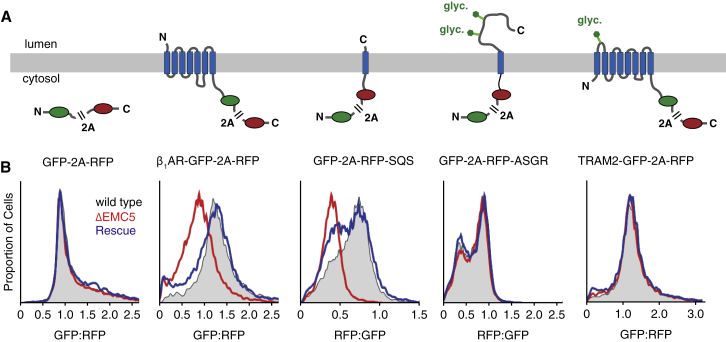
EMC Is Required for Optimal β_1_AR Biogenesis in Cells (A) Diagram and topology of constructs for analysis of protein biogenesis by flow cytometry. All constructs contain GFP and RFP separated by a viral 2A peptide that mediates peptide bond skipping. Changes in the stability of a test protein fused to one of the fluorescent proteins changes the GFP:RFP fluorescence ratio. (B) Histograms of flow cytometry data monitoring the fluorescence protein ratio in the indicated U2OS cell lines for each construct. “ΔEMC5” indicates a knockout of EMC5, while “rescue” indicates ΔEMC5 cells rescued by inducible re-expression of a stably integrated EMC5. See also Figure S1.

Among the several membrane proteins reported to be impacted by EMC disruption, we chose to analyze GPCRs. Earlier analysis had placed EMC’s role at an early stage of a GPCR’s functional expression in *Drosophila* (Satoh et al., 2015) but could not distinguish between effects on translation, maturation, degradation, or trafficking. To investigate this, we analyzed post-translational effects of EMC disruption on the vertebrate β_1_-adrenergic receptor (β_1_AR) using a flow cytometry-based assay. The C terminus of a well-characterized β_1_AR construct (Warne et al., 2009) was appended with GFP and RFP separated by a viral P2A sequence (Figure 1A). Translation of this mRNA will generate two products due to peptide bond skipping at the P2A sequence (de Felipe et al., 2006): the β_1_AR-GFP fusion protein and a separate RFP. Thus, metabolically stable RFP serves as a “counter” for the number of times this construct is translated, effectively integrating mRNA levels and translation efficiency into a single metric. Because one β_1_AR-GFP is synthesized for each RFP, any reduction in GFP levels relative to RFP necessarily reflects post-translational degradation.

**Figure S1:**
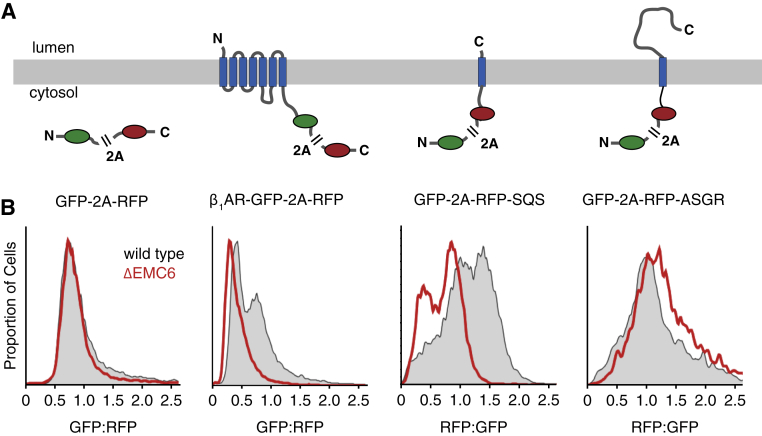
EMC Is Required for Optimal β_1_AR Biogenesis in Cells, Related to Figure 1 (A) Diagram and topology of constructs for analysis of protein biogenesis by flow cytometry. All constructs contain GFP and RFP separated by a viral 2A peptide that mediates peptide bond skipping. Changes in the stability of a test protein fused to one of the fluorescent proteins changes the GFP:RFP fluorescence ratio. (B) Histograms of flow cytometry data monitoring the fluorescence protein ratio in unmodified (wild-type) or EMC6-disrupted (ΔEMC6) HEK293 TREX cells.

Relative to the baseline distribution of GFP:RFP ratios for the β_1_AR reporter in wild-type U2OS cells, the distribution was clearly reduced (by ∼2-fold) in cells lacking EMC5 (Figure 1B), a core subunit of EMC whose deletion eliminates the entire complex (Guna et al., 2018). Similar results were obtained in HEK293 cells disrupted for EMC6 (Figure S1), a different core EMC subunit essential for integrity of the entire complex. Acute reintroduction of EMC5 via an inducible promoter in EMC5-knockout cells restored the complete EMC (Guna et al., 2018) and completely rescued the reduced stability of the β_1_AR reporter. Very similar effects of EMC disruption were observed for the tail-anchored protein squalene synthase (Figure 1B), a protein whose insertion into the ER is established to be EMC-mediated (Guna et al., 2018). Reporter cassettes lacking an insert or containing the cell surface protein asialoglycoprotein receptor (ASGR1) or the ER-resident protein TRAM2 showed no GFP:RFP ratio changes in EMC-knockout or rescue cells relative to wild-type cells.

These results indicate that post-translational β_1_AR stability is dependent on EMC, the absence of which leads to its elevated degradation. The absence of any appreciable effect on either ASGR1 or TRAM2 excludes non-specific perturbation of protein biosynthesis or trafficking. More specifically, the core steps of SRP-dependent targeting and Sec61-dependent insertion, both of which are essential for optimal ASGR1 biogenesis (Görlich and Rapoport, 1993, Spiess and Lodish, 1986), are all apparently normal in EMC-disrupted cells.

### Reconstitution of EMC-Dependent β_1_AR Biogenesis *In Vitro*

The altered β_1_AR stability in EMC-disrupted cells is compatible with several explanations including altered biogenesis, trafficking, promiscuous degradation, and others. To investigate β_1_AR biogenesis, we used an *in vitro* translation system composed of reticulocyte lysate and ER-derived rough microsomes (RMs). This system recapitulates membrane protein insertion, but is not confounded by post-translational degradation or vesicular trafficking out of the ER.

**Figure S2:**
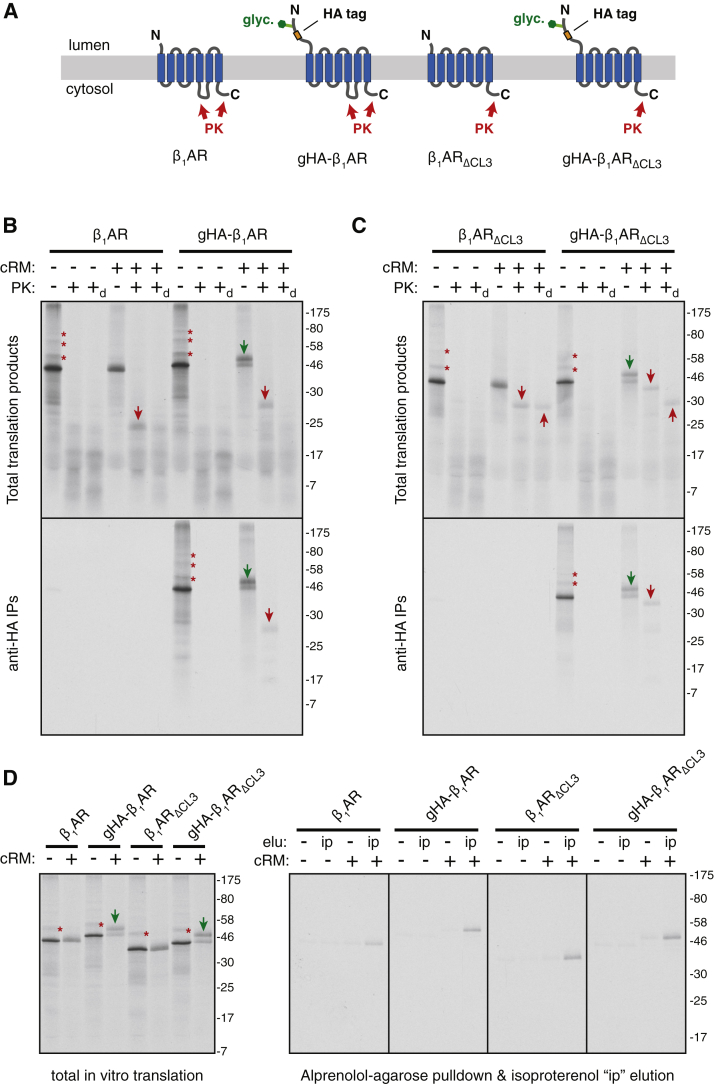
Reconstitution of EMC-Dependent β_1_AR Biogenesis *In Vitro*, Related to Figure 2 (A) Diagram of constructs used to characterize β_1_AR topogenesis. ΔCL3 refers to the shortening of the cytosolic loop 3 between TMD5 and TMD6. The sites that should be accessible to cytosolically added proteinase K (PK) are indicated for each construct. (B and C) ^35^S-methionine labeled β_1_AR (or one of the indicated variants) was translated in reticulocyte lysate (RRL) in the absence or presence of canine pancreas-derived rough microsomes (cRM). The translation products were either left untreated or digested with proteinase K without or with detergent (subscripted d) as indicated. The samples were either analyzed directly (total translation products) or after immunoprecipitation via the N-terminal HA tag (anti-HA IPs) and analyzed by SDS-PAGE and autoradiography. Asterisks indicate ubiquitinated products; green arrows indicate glycosylated products; red downward arrows indicate PK-protected N-terminal fragments; red upward arrows indicate the protease-resistant 7-TMD core of β_1_AR left after digestion of the N- and C-terminal tails in the ΔCL3 variants. These assigned identities of the bands can be deduced by a combination of their size, change in migration upon addition of the N-terminal glycosylation site, change in digestion pattern upon shortenting of CL3 to make it proteaese-inaccessible, and IP via the HA epitope. (D) ^35^S-methionine labeled β_1_AR (or one of the indicated variants) was translated in RRL in the absence or presence of microsomes (cRM). An aliquot of the sample was analyzed directly (total *in vitro* translation) or solubilized and incubated with immobilized alprenolol (a β_1_AR antagonist). The resin was washed, then eluted in buffer without or with isoproterenol (ip; a β_1_AR agonist). Efficient recovery is only observed when β_1_AR is synthesized with cRM and eluted with isoproterenol.

In preliminary experiments, we established the conditions and assays to monitor membrane insertion, topology, and folding of newly synthesized ^35^S-methionine-labeled β_1_AR (Figure S2). Correct topogenesis was inferred by a combination of glycosylation, selective accessibility to cytosolically added protease, and immunoprecipitation (Figures S2A–S2C). These results demonstrated that ∼50%–60% of β_1_AR could be inserted properly in this system. Furthermore, the inserted population appears to be capable of efficient folding as judged by the acquisition of protease resistance of the core 7-TMD domain even in the presence of detergent (Figure S2C). Consistent with this interpretation, the inserted population binds to immobilized alprenolol, a β_1_AR antagonist, and selectively elutes with the agonist isoproterenol (Figure S2D).

**Figure 2:**
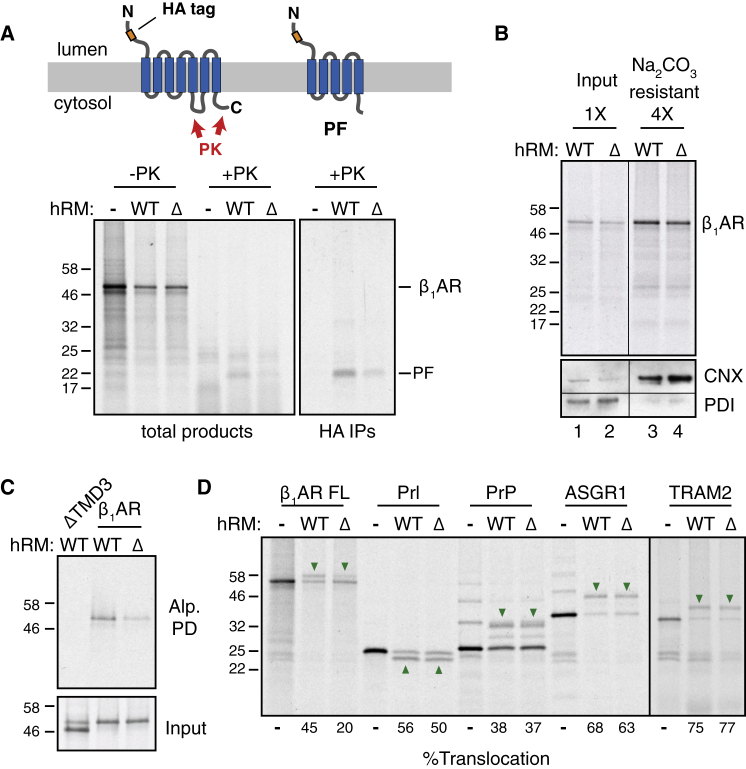
Reconstitution of EMC-Dependent β_1_AR Biogenesis *In Vitro* (A) ^35^S-methionine labeled β_1_AR was translated in reticulocyte lysate (RRL) in the absence or presence of HEK293-derived rough microsomes (hRM) from wild-type (WT) or ΔEMC6 (Δ) cells. The translation products were digested with proteinase K (+PK) or left untreated (−PK), then analyzed directly (total products) or after immunoprecipitation via the HA epitope tag (HA IPs). The positions of full-length β_1_AR and the protease-protected fragment (PF) are indicated. The sites accessible to PK and the resulting PF are shown in the diagram above the gel. (B) ^35^S-methionine labeled β_1_AR translation products produced in WT or ΔEMC6 hRMs were isolated by sedimentation of the hRMs and analyzed directly (input) or after extraction with Na_2_CO_3_ at pH 11.5 (Na_2_CO_3_ resistant; 4-fold excess was analyzed). β_1_AR was visualized by autoradiography, while the integral membrane ER protein calnexin (CNX) and ER-lumenal protein disulfide isomerase (PDI) were detected by immunoblotting. (C) ^35^S-methionine labeled β_1_AR or a mutant lacking the third transmembrane domain (ΔTM) were tested for binding to immobilized alprenolol. The starting translation products (input) and alprenolol pull-downs (Alp. PD) are shown. (D) The indicated proteins were translated without or with the indicated hRMs and analyzed for translocation by their glycosylation (downward green arrows) or signal peptide cleavage (upward green arrows). The % glycosylated or signal cleaved was quantified and shown below the gel. See also Figures S2 and S3.

Using these assays, we assessed the consequences of EMC-disruption by using RMs derived from wild-type (WT) versus EMC6-knockout (ΔEMC6) HEK293 cells. The protease-protected N-terminal fragment diagnostic of correct topogenesis of the first five TMDs (Figure S2B) was reduced by more than 50% in RMs from ΔEMC6 cells relative to wild-type cells (Figure 2A). Importantly, equal amounts of β_1_AR were recovered in membranes pelleted from these two reactions (Figure 2B, lanes 1 and 2) and were similarly resistant to alkaline extraction (Figure 2B, lanes 3 and 4). Furthermore, β_1_AR in ΔEMC6 microsomes was less efficiently captured by immobilized alprenolol ligand than β_1_AR in wild-type microsomes (Figure 2C), while a folding-deficient construct (ΔTM3) was not recovered at all. These results suggest that although β_1_AR is inserted into ΔEMC6 microsomes with comparable efficiency to wild-type microsomes, it is impaired in reaching a topologically correct ligand-binding state.

**Figure S3:**
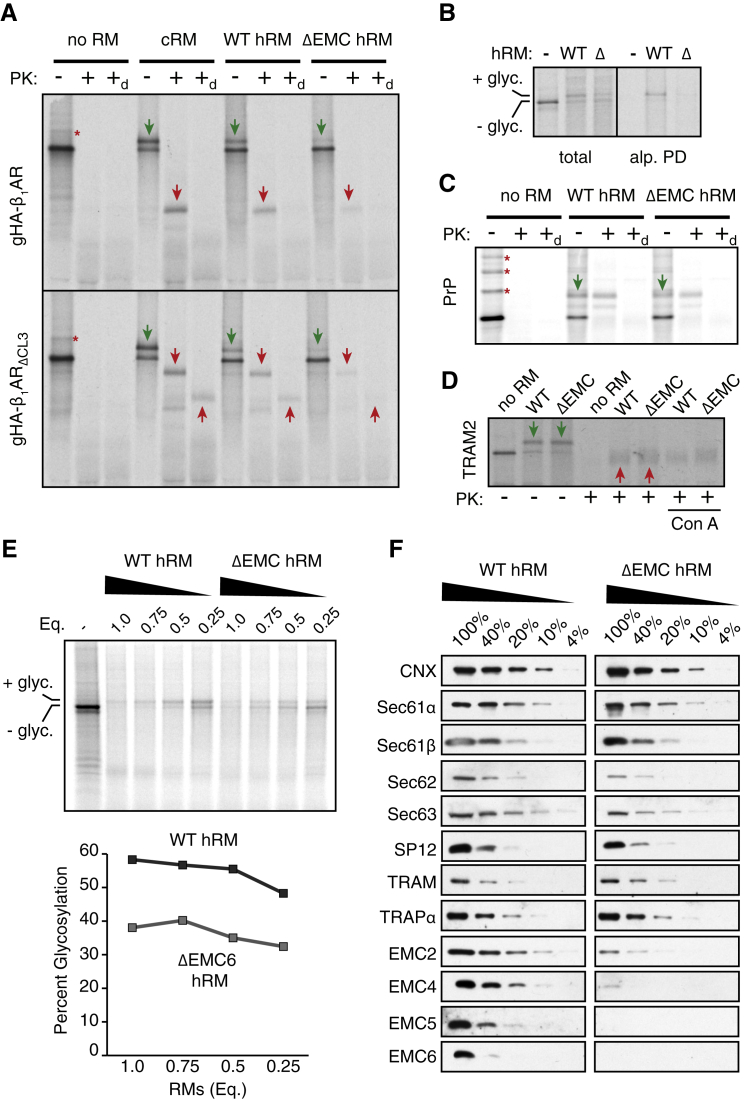
Reconstitution of EMC-Dependent β_1_AR Biogenesis *In Vitro*, Related to Figure 2 (A) Protease protection assay on the indicated constructs performed as in Figure S2B, but with either cRM or HEK293-derived microsomes (hRM) from either wild-type (WT) or ΔEMC6 (ΔEMC) cells. Asterisks indicate ubiquitinated products; green arrows indicate glycosylated products; red downward arrows indicate PK-protected N-terminal fragments; red upward arrows indicate the protease-resistant 7-TMD core of β_1_AR left after digestion of the N- and C-terminal tails in the ΔCL3 variants. (B) ^35^S-methionine labeled gHA-β_1_AR was translated in reticulocyte lysate (RRL) in the presence of wild-type (WT) or ΔEMC6 (Δ) hRM. The samples were analyzed directly (total) or after selective capture by immobilized alprenolol and elution with isoproterenol (alp. PD). (C) ^35^S-methionine labeled mammalian prion protein (PrP) was translated without or with the indicated hRM and analyzed by the PK-protection assay. Asterisks indicate ubiquitinated products; green arrows indicate doubly-glycosylated products (PrP contains two glycosylation sites). (D) ^35^S-methionine labeled human TRAM2 was translated without or with the indicated hRM and analyzed by the PK-protection assay. Green arrows indicate TRAM2 glycosylated in the loop between the first and second TMD (see Figure 1A). After protease digestion, only the cytosolic-facing N- and C-terminal ends of the protein are digested, leaving behind a folded core (upward red arrows) comprising all eight TMDs. This product is recovered with ConA, verifying that it is the glycosylated central core. Note that no difference in TRAM2 glycosylation or protease-protection is seen between reactions performed with hRM from wild-type or ΔEMC cells. (E) ^35^S-methionine labeled gHA-β_1_AR was translated in RRL without or with wild-type (WT) or ΔEMC6 (Δ) hRM at various relative concentrations (WT and ΔEMC hRM were normalized to have equal total protein concentration as judged by absorbance at 280 nm). The samples were analyzed directly (top panel) and the percent of translation product that is glycosylated was quantified by phosphorimager and plotted (bottom panel). (F) Different relative amounts of WT or ΔEMC6 hRM were analyzed by immunoblotting for the indicated ER-resident proteins. Note that the WT and ΔEMC6 samples that are being compared were analyzed on the same gel and processed together.

Similar results were obtained for both non-glycosylated and glycosylated versions of β_1_AR (Figure 2A versus S3A, respectively), and regardless of whether folding was assessed by ligand binding (Figure 2C, S3B) or protease-protection of the 7-TMD core (Figure S3A). In each case, successful biogenesis was reduced by more than 50% in ΔEMC6 RMs and this was consistently observed across multiple independent microsome preparations. Impaired biogenesis of β_1_AR in ΔEMC6 RMs was accompanied by reduced glycosylation (Figures S3A and S3B), despite the fact that glycosylation of other proteins in these same microsomes was unaffected (Figures S3C and S3D). The biogenesis deficiency in ΔEMC6 RMs could not be overcome by using more microsomes in the reaction (Figure S3E), further arguing for an intrinsic problem in making β_1_AR correctly in the absence of EMC.

Using glycosylation, signal peptide cleavage, and protease protection assays, we found that biogenesis of the secretory protein prolactin, the GPI-anchored prion protein (PrP), the single-pass membrane protein ASGR1, and the multi-pass membrane protein TRAM2 were unaffected in ΔEMC6 microsomes (Figures 2D, S3C, and S3D, and data not shown). These substrates represent the major types of model proteins analyzed in earlier work and collectively report on the integrity of SRP-dependent targeting, Sec61-mediated translocation and membrane insertion, the modulatory functions of known translocon accessory factors, and the enzymatic activities of signal peptidase and OST. Indeed, immunoblotting verified that these components do not differ appreciably between wild-type and ΔEMC6 microsomes (Figure S3F). Thus, the selective β_1_AR biogenesis defect observed in EMC-deficient cells (Figure 1) can be recapitulated *in vitro*. Furthermore, the observation that glycosylation of an acceptor site near the N terminus of β_1_AR is diminished in ΔEMC6 microsomes suggested that a relatively early step of β_1_AR biogenesis may be impaired. Although we have not further characterized the mis-inserted forms of β_1_AR in ΔEMC6 microsomes, they appear to be recognized by the cell’s quality control systems and degraded.

### EMC Is Required for Accurate TMD1 Topogenesis of β_1_AR

**Figure S4:**
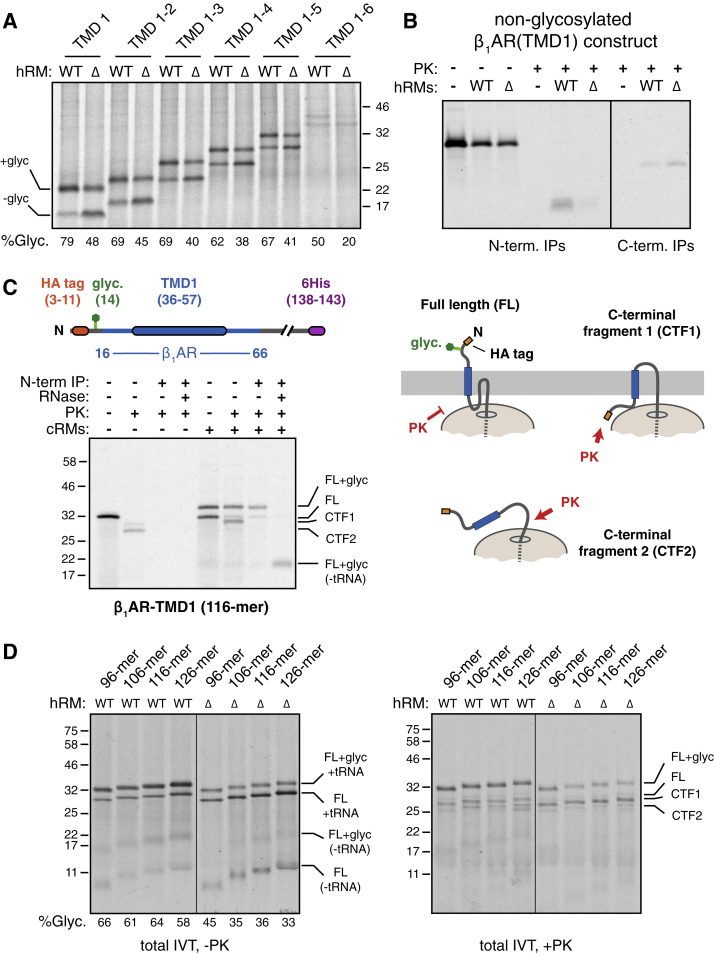
EMC Is Required for Accurate TMD1 Topogenesis of β_1_AR, Related to Figure 3 (A) ^35^S-methionine labeled gHA-β_1_AR constructs terminated after the indicated number of TMDs was translated in reticulocyte lysate (RRL) in the presence of wild-type (WT) or ΔEMC6 (Δ) hRM. The samples were analyzed directly (total) and the proportion of polypeptide that is glycosylated was quantified by phosphorimaging. (B) An experiment similar to Figure 3A was performed with a construct lacking the N-terminal glycosylation site. (C) 116-residue ribosome-nascent chain complexes of gHA-β_1_AR (see diagram) truncated 60 residues beyond the TMD were produced in RRL. They were incubated without or with canine pancreas-derived microsomes (cRMs) and subjected to digestion with proteinase K (PK) as indicated. An aliquot of the PK-digested sample was subsequently immunoprecipitated via the N-terminal HA tag without or with RNase digestion as indicated.The diagram to the right shows the interpretation of the different products: N_exo_-inserted nascent chains are glycosylated and fully protected from PK; non-inserted nascent chains are non-glycosylated and accessible to PK outside the ribosome and generate a C-terminal fragment (CTF2); N_cyt_ nascent chains are also non-glycosylated and accessible to PK, but have some regions protected by the membrane to generate a slightly larger C-terminal fragment (CTF1). (D) Ribosome-nascent chain complexes of gHA-β_1_AR truncated at the indicated lengths were produced in RRL, incubated with wild-type (WT) or ΔEMC6 (Δ) hRM, and analyzed directly (total IVT, -PK) or subjected to digestion with proteinase K (PK) before analysis (total IVT, +PK). The products are labeled as in (C).

**Figure 3:**
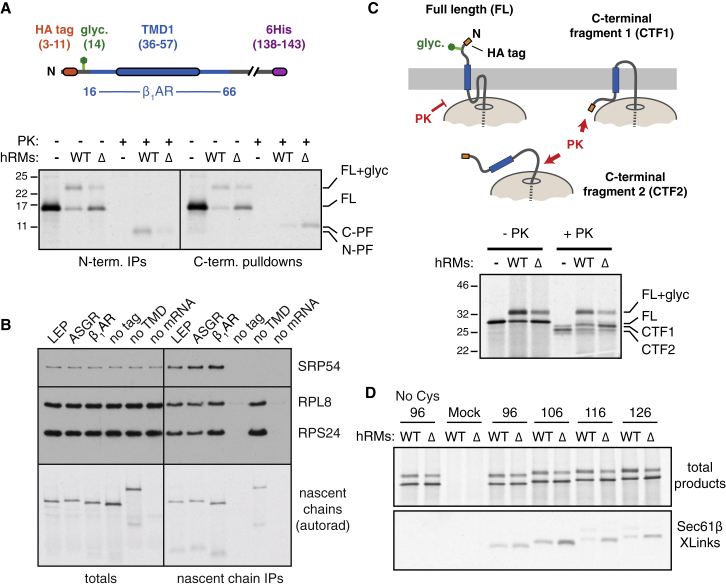
EMC Is Required for Accurate TMD1 Topogenesis of β_1_AR (A) ^35^S-methionine labeled β_1_AR-TMD1 (shown in the diagram) was translated in the absence or presence of WT or ΔEMC6 (Δ) hRMs, subjected to PK digestion as indicated, and the products recovered by either immunoprecipitation via the N-terminal HA tag (N-term. IPs) or pull-downs via the C-terminal His6 tag (C-term. pull-downs). The positions of unmodified full-length (FL) product, glycosylated product (+glyc), and N- and C-terminal protease-protected fragments (N-PF and C-PF, respectively) are indicated. (B) ^35^S-methionine labeled ribosome-nascent chains (stalled 39 residues downstream of the indicated TMDs) produced in reticulocyte lysate were affinity purified via an N-terminal FLAG epitope tag and analyzed by autoradiography to detect the nascent chains or immunoblotting for ribosomal proteins (RPL8 and RPS24) and SRP54. Controls either lacked an epitope tag, TMD, or mRNA. (C) ^35^S-methionine labeled 116-residue nascent chains of β_1_AR were targeted to WT or ΔEMC6 hRMs and analyzed by the PK protection assay. The diagram indicates which species are glycosylated and PK-resistant versus non-glycosylated and PK-accessible. (D) ^35^S-methionine labeled β_1_AR nascent chains of the indicated lengths were targeted to WT or ΔEMC6 hRMs (top panel), then subjected to sulfhydryl-mediated crosslinking. The crosslinked products were immunoprecipitated using antibodies against Sec61β and shown in the bottom panel. Controls lacking either mRNA (mock) or a cysteine in the nascent chain showed no Sec61β immunoprecipitated products. See also Figure S4.

To facilitate the analysis of early events in β_1_AR biogenesis, we sought a simplified construct that still showed EMC-dependence. Serial truncations of β_1_AR from the C terminus revealed that the glycosylation defect was retained even in a construct that only contained the first TMD (Figure S4A). This simplified construct (termed β_1_AR-TMD1) was effectively glycosylated at the N terminus in wild-type microsomes, but impaired by more than ∼50% in ΔEMC microsomes (Figure 3A). Protease digestion produced a protected fragment recovered by immunoprecipitation via a N-terminal HA epitope tag. As expected from the glycosylation analysis, this N-terminal fragment was substantially reduced in matched reactions containing ΔEMC6 microsomes. Thus, insertion of β_1_AR-TMD1 in the correct topology is EMC-dependent, recapitulating the EMC-dependence of full-length β_1_AR *in vivo* and *in vitro*.

Unexpectedly, pull-downs of the same samples via a C-terminal His6-tag revealed a protease-protected fragment preferentially in the ΔEMC6 samples (Figure 3A). This fragment was also seen at low levels in wild-type samples. Importantly, no protease protected fragments were observed in samples lacking RMs. This suggests that both wild-type and ΔEMC6 microsomes are comparably efficient in β_1_AR-TMD1 insertion, consistent with the resistance to alkaline extraction of full-length β_1_AR (Figure 1B). However, the topology of around half of β_1_AR-TMD1 molecules is inverted in ΔEMC6 microsomes, explaining the impaired N-terminal glycosylation of various β_1_AR constructs (Figure S4A). A version of β_1_AR-TMD1 lacking the N-terminal glycosylation site also showed topologic inversion in ΔEMC6 microsomes (Figure S4B), arguing against glycosylation influencing topogenesis.

To determine the point at which topogenesis diverges, we turned to the analysis of ribosome-nascent chain complexes (RNCs) of different lengths representing intermediates in the targeting and insertion of β_1_AR-TMD1. As expected for a signal anchor sequence (Sakaguchi et al., 1987, Spiess and Lodish, 1986), 96-residue long cytosolic RNCs of β_1_AR-TMD1 were associated with SRP similarly to the previously established N_exo_ and N_cyt_ model membrane proteins LepB (leader peptidase from *E. coli*) and ASGR1, respectively. Protease digestion of such RNCs removes the exposed N terminus, leaving behind a tRNA-associated C-terminal fragment protected by the ribosome (Figures 3C and S4C). A minor, slightly larger product may either represent partial protection by SRP, or some heterogeneity in the precise site of protease digestion.

When 116-residue long β_1_AR-TMD1 RNCs are presented to RMs, translocation of the N terminus enables glycosylation, and this product is fully shielded from cytosolic protease by the ribosome and membrane (Figures 3C and S4C). Relative to the situation in wild-type microsomes, ΔEMC6 microsomes show less glycosylation and less full-length protease protection (Figure 3C). Instead, there is increased amounts of a non-glycosylated product whose N terminus is accessible to protease. Because the protected fragment is slightly larger than that seen in the absence of microsomes, it appears that the membrane affords protection of ribosome-proximal regions of the nascent chain.

This difference in insertion between wild-type and ΔEMC6 microsomes is observed across a range of nascent chain lengths. Of note, the difference was not as prominent for the 96-residue RNC that is truncated only 39 residues beyond the TMD (Figures 3D, top, and S4D). At this length, the TMD has barely emerged from the ribosome and is just long enough for membrane insertion in the N_exo_ topology, but cannot achieve the N_cyt_ orientation. Thus, the EMC requirement can apparently be partially bypassed by constraining the RNC to only the N_exo_ option and providing far more time for insertion than would be available during co-translational biogenesis of full-length β_1_AR. The fact that even this highly biased situation still shows an appreciable difference in insertion suggests that the deficiency observed in ΔEMC6 microsomes is not simply a kinetic problem; rather, the microsomes are intrinsically less capable of TMD1 insertion in the N_exo_ topology.

Chemical crosslinking of RNCs via a cysteine preceding the TMD validated the conclusions from the protease protection assay. We monitored crosslinks between the nascent chain and a single cytosolic cysteine in Sec61β to assess the cytosolic disposition of sequences preceding the TMD. At each length, crosslinking to Sec61β was greater in ΔEMC6 microsomes than matched wild-type reactions (Figure 3D, bottom). This is the mirror image of the extent of glycosylation in these same samples (Figure 3D, top) because cysteine availability in the cytosol is mutually exclusive with glycosylation of an acceptor site four residues away. This indicates that RNCs that fail successful N_exo_ insertion in ΔEMC6 microsomes are at the Sec61 translocon with the N terminus facing the cytosol. For the reasons articulated above, this difference is less prominent for the 96-residue RNCs. Taken together, the findings with β_1_AR-TMD1 suggest that nascent β_1_AR normally engages SRP, targets to the ER, and inserts in the N_exo_ orientation in a reaction that is stimulated by EMC. In the absence of EMC, N_exo_ insertion is less efficient, resulting in the non-inserted β_1_AR being near the Sec61 translocon.

### TMD1 of Most GPCRs Requires EMC for Optimal Insertion

**Figure 4:**
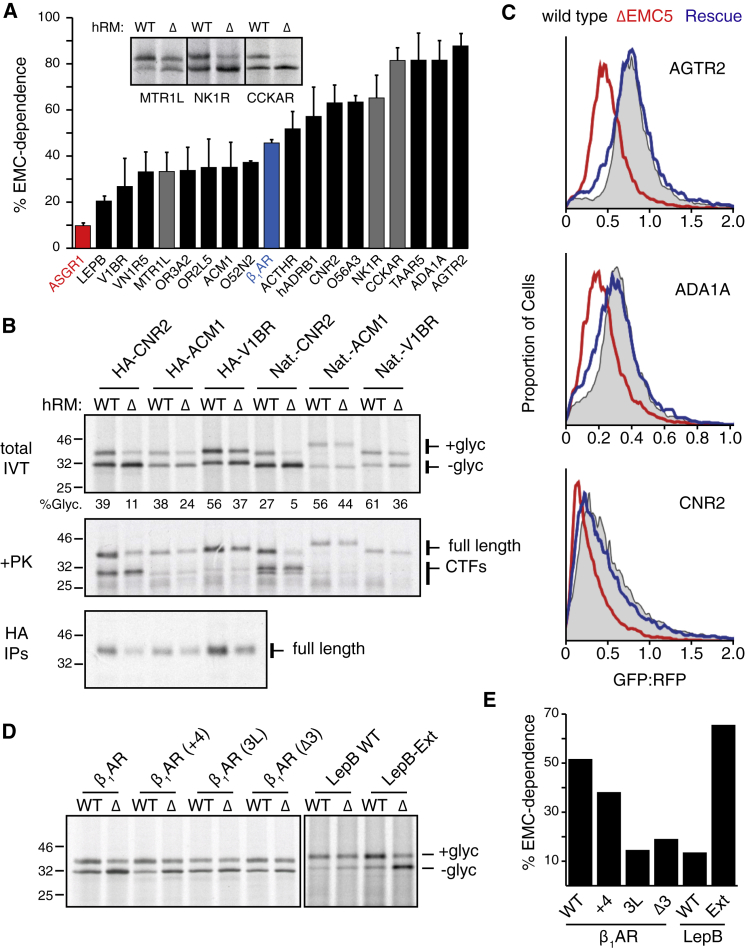
TMD1 of Most GPCRs Requires EMC for Optimal Insertion (A) Constructs containing TMD1 and flanking regions from the indicated GPCRs (see Table S1) were analyzed by glycosylation of nascent chains targeted to WT or ΔEMC6 (Δ) hRMs. The % decrease in ΔEMC6 hRM was quantified from three experiments and plotted and error bars represent standard deviation from the mean. Example data from the three GPCRs indicated by gray bars are shown in the inset. The model proteins ASGR1 and LepB were also analyzed for EMC-dependence and plotted for comparison. (B) Ribosome-nascent chains (stalled ∼60 residues downstream of the indicated TMDs) were targeted to WT or ΔEMC6 (Δ) hRMs and analyzed by the PK-protection assay as in Figure 3C. “HA” indicates an N-terminal HA tag and glycosylation site (see Figure 3A), while “Nat.” indicates the native N-terminal domain. The PK-digested samples from the HA-containing constructs were also subjected to immunoprecipitation (HA IPs). (C) The indicated GPCRs were tagged as in Figure 1A and analyzed by flow cytometry as in Figure 1B. Grey trace is WT cells, red trace is ΔEMC5 cells, and blue trace is EMC5-rescued ΔEMC5 cells. (D) Ribosome-nascent chains of the indicated constructs (Table S2) were analyzed for insertion by the glycosylation assay using WT and ΔEMC6 hRMs. (E) Quantification of the autoradiograph shown in (D). See also Figure S5 and Tables S1 and S2.

To determine whether the first TMDs of other GPCRs also rely on EMC, we analyzed constructs containing TMD1 and flanking regions of sixteen GPCRs (Table S1) in a context similar to β_1_AR-TMD1 (Figure 3A). Using glycosylation of an N-terminal site in 116-residue RNCs as the readout, we found that all GPCRs tested showed at least a partial dependence on EMC, ranging from ∼20% to over 90% impairment in its absence (Figure 4A). This conclusion from glycosylation analysis was verified by protease protection assays and N-terminal immunoprecipitation (three examples are shown in Figure 4B). Importantly, analysis of RNC intermediates for three native GPCR N-terminal sequences showed a similar degree of impaired insertion in ΔEMC6 microsomes as seen for the respective epitope-tagged TMD1 constructs (Figure 4B). Thus, early events in the biogenesis of most GPCRs differs at least partially in EMC-deficient microsomes *in vitro*. Consistent with the lack of effect in cells (Figure 1A), ASGR1 showed little or no deficiency in insertion into ΔEMC6 microsomes, while LepB showed a very small but reproducible EMC-dependence (Figure 4A).

Three full-length GPCRs were analyzed in U2OS cells for impaired biogenesis using the dual-color flow cytometry assay (Figure 4C). As expected for expression in a heterologous cell type, the steady-state levels of each receptor varied somewhat, with the type 2 angiotensin II receptor (AGTR2) expressing better than either α_1A_-adrenergic receptor (ADA1A) or Cannabinoid receptor 2 (CNR2). Nevertheless, the steady-state level of the GFP-tagged GPCR was reduced in ΔEMC5 cells for each protein, but restored to wild-type levels when EMC5 was re-expressed. Thus, the insertion impairment of TMD1 seen in ΔEMC6 microsomes *in vitro* corresponds to reduced post-translational stability of the full GPCR in ΔEMC5 cells.

**Figure S5:**
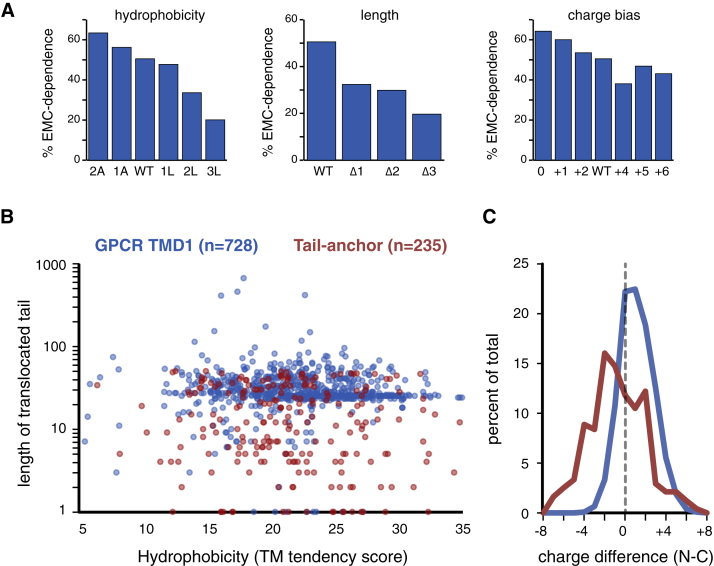
Properties of TMD1 from GPCRs Analyzed in This Study, Related to Figure 4 (A) The indicated β_1_AR constructs (see Table S2) were tested for insertion into wild-type and ΔEMC hRM as in Figure 4A. Glycosylation was used to quantify the amount of insertion in the correct (N_exo_) orientation. The relative difference in correct insertion between wild-type and ΔEMC microsomes was used to determine EMC-dependence (i.e., 60% insertion in ΔEMC relative to wild-type would mean 40% EMC-dependence). All of the constructs were analyzed together. The wild-type is re-plotted in each of the three graphs for comparison. Note that EMC-dependence of the β_1_AR TMD is influenced by hydrophobicity, TMD length, and to a lesser extent, flanking charge bias. (B) Plot of TM tendency score versus length of the translocated domain for all non-signal-containing GPCRs and ER-localized TA proteins in the human genome. The translocated domain of almost all TA and N_exo_ signal anchors is less than ∼40 residues. (C) Histogram of the charge difference for the dataset in (B). Note that in both cases, there is a slight preference for net positive charges facing the cytosol.

The TMDs that display EMC-dependence (whether partial or near-complete) are diverse in hydrophobicity, flanking charges, length, and amino acid composition (Table S1). To determine which feature(s) influence EMC-dependence, we analyzed the insertion of various β_1_AR-TMD1 mutants (Table S2). We found that β_1_AR-TMD1 could be made less EMC-dependent by reducing its length, increasing its hydrophobicity, or biasing the flanking charge distribution to favor cytosolic basic residues (Figures 4D, 4E, and S5A). Conversely, lengthening the LepB TMD with three non-hydrophobic residues made it strongly EMC-dependent (Figures 4D and 4E). These observations partially explain the variable EMC dependence of natural N_exo_ signal anchors (e.g., Figure 4A), although a fully predictive algorithm will require extensive analysis analogous to studies of Sec61-mediated insertion (Hessa et al., 2007).

### N_exo_ Signal Anchor Insertion Can Occur without the Sec61 Complex

The only factor previously implicated in signal anchor insertion is the Sec61 complex (Heinrich et al., 2000, High et al., 1993, Oliver et al., 1995). However, a strict requirement for Sec61 in depletion experiments has only been shown for the secretory protein prolactin (Görlich and Rapoport, 1993). The recently demonstrated insertase activity of EMC (Guna et al., 2018) led us to hypothesize that the observed EMC-dependence of various N_exo_ signal anchors might be explained by their direct insertion via EMC. In support of this idea, it is noteworthy that tail-anchors inserted by EMC are similar to N_exo_ signal anchors in having relatively short translocated domains (Figure S5B) and basic residues enriched on the cytosolic flank of the TMD (Figure S5C). If EMC were the insertase for N_exo_ signal anchors, Sec61 might be dispensable for this event similar to the Sec61-independence of tail-anchor insertion.

**Figure 5:**
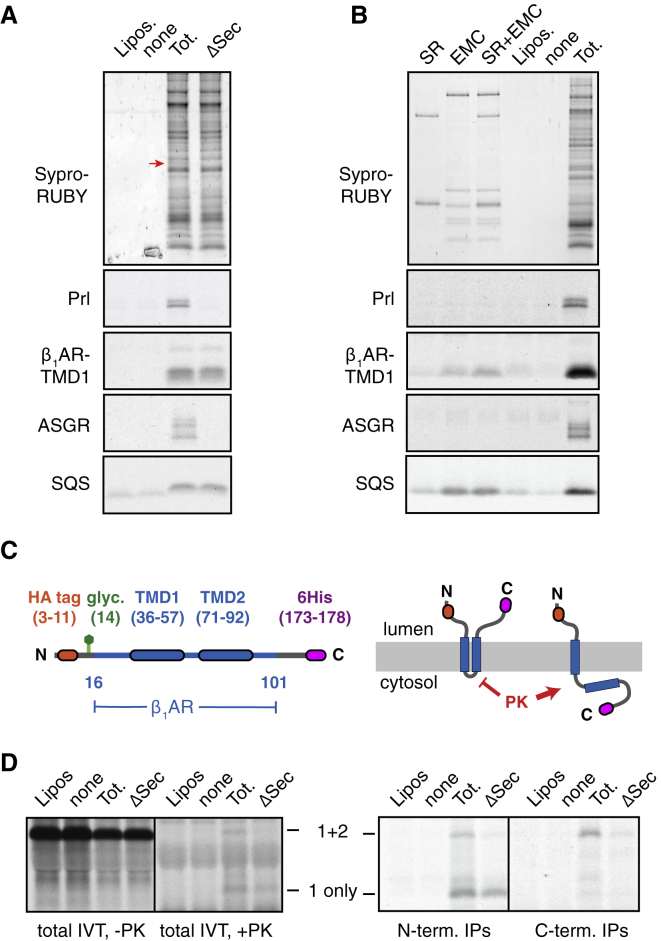
EMC and Sec61 Complex Act at Different Steps during β_1_AR Insertion (A) The indicated constructs were analyzed by the protease-protection assay for translocation into liposomes (Lipos) or proteoliposomes reconstituted from total ER proteins (Tot.) or ER proteins immunodepleted of the Sec61 complex (ΔSec). Total proteins in the proteoliposomes were visualized by Sypro Ruby, with the position of Sec61α indicated by the red arrow. Depletion was verified to be over 95% (see Figure S6A). The remaining panels show protease-protected (and hence, translocated) products recovered by immunoprecipitation. (B) The indicated constructs were analyzed by the protease-protection assay for translocation into liposomes (Lipos), proteoliposomes reconstituted from total ER proteins (Tot.), or proteoliposomes containing the indicated purified proteins (SR is SRP receptor). Proteins in the proteoliposomes were visualized by Sypro Ruby. 10-fold excess of the first four lanes were loaded to detect the purified proteins. EMC and SR did not contain any detectable Sec61 contamination (see Figure S6D). The remaining panels show protease-protected (and hence, translocated) products recovered by immunoprecipitation. (C) Diagram of the two-TMD β_1_AR construct and its topology when TMD2 inserts or fails to insert into the membrane. Only the single-spanning form is accessible to proteinase K (PK) digestion (see Figure S7). (D) The two-TMD construct from (C) was analyzed in the indicated proteoliposome preparations by the protease-protection assay. The left panel shows total products, while the right panel shows the PK-digested products after recovery via N- or C-terminal tags as indicated. “1+2” indicates the protected product indicative of the double-spanning topology, and “1 only” indicates the single-spanning topology. See also Figures S6 and S7.

**Figure S6:**
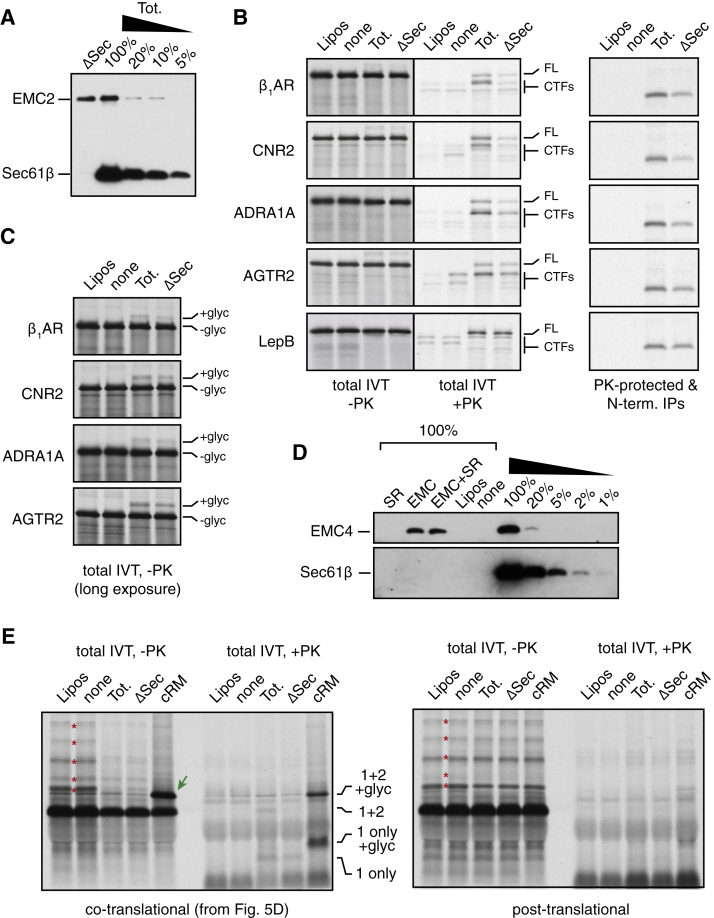
EMC and Sec61 Complex Act at Different Steps during β_1_AR Insertion, Related to Figure 5 (A) Immunoblotting of proteoliposomes (PLs) reconstituted from total ER proteins (Tot.) or Sec61-depleted ER proteins (ΔSec) shows that under conditions where even 5% of total PLs show readily detectable Sec61, none is seen in ΔSec PLs. EMC levels are comparable. (B) Ribosome-nascent chain complexes of constructs containing the indicated TMD1 regions (see diagram, Figure S5A) truncated ∼60 residues beyond the TMD (corresponding to residue 116 in the β_1_AR-TMD1 construct) were produced in RRL. They were incubated without anything, with liposomes, or with PLs from total ER proteins (Tot.) or Sec61-depleted ER proteins (ΔSec). An aliquot of the sample was analyzed directly (-PK) or subjected to digestion with proteinase K (+PK). An aliquot of the PK-digested sample was subsequently immunoprecipitated via the N-terminal HA tag after RNase digestion (N-term. IPs). FL indicates full length product protected from protease, indicative of successful insertion. CTFs indicate C-terminal fragments from non-inserted products. (C) The total IVT products from panel B shown from an overexposed autoradiograph to visualize the minor glycosylated product (+glyc). Glycosylation is relatively inefficient in PLs compared to native microsomes. (D) The PLs from Figure 5B were analyzed by immunoblotting for Sec61 and EMC to verify no Sec61 contamination of either EMC or SRP receptor (SR) PLs. (E) The two-TMD β_1_AR construct (see Figure 5C) was analyzed in the indicated proteoliposome preparations or canine-pancreas derived microsomes (cRM) by the protease-protection assay. Samples were analyzed directly without immunoprecipitation. The left panel shows the experiment when membranes are present during the translation reaction (co-translational; reproduced from Figure 5D), while the right panel shows the experiment when incubation with membranes was post-translational. Red asterisks indicate ubiquitinated products, green arrow indicates the glycosylated product, “1+2” indicates the protected product indicative of the double-spanning topology, and “1 only” indicates the single-spanning topology.

To investigate this hypothesis, we examined GPCR TMD1 insertion into membranes depleted of the Sec61 complex. RMs were solubilized, incubated without or with immobilized antibodies against the Sec61 complex, and the unbound proteins reconstituted into proteoliposomes (PLs). Sec61 was verified to be thoroughly depleted (by over 95%; Figure S6A), while the overall protein profile was otherwise unchanged (Figure 5A). As shown previously (Görlich and Rapoport, 1993), Sec61-depleted PLs are completely deficient in prolactin translocation (Figure 5A). Furthermore, they cannot detectably insert the N_cyt_ signal anchored protein ASGR1 as measured by protease protection assays.

Remarkably, however, several different N_exo_ signal anchored proteins from GPCRs can be inserted into Sec61-depleted PLs. Protease-protection assays and immunoprecipitations (IPs) via an N-terminal tag showed that the N terminus is protected from digestion in nondepleted and Sec61-depleted PLs, but not empty liposomes (Figures 5A and S6B). Importantly, the extensively studied model protein LepB whose insertion was thought to require the Sec61 complex (Heinrich et al., 2000) was inserted almost equally well in non-depleted or depleted PLs (Figure S6B). Although glycosylation is relatively inefficient in PLs, over-exposed autoradiographs showed that an N-terminal acceptor site is glycosylated comparably efficiently for several different N_exo_ signal anchors in both nondepleted and Sec61-depleted PLs (Figure S6C).

While insertion of some of these signal anchors was reduced by ∼50% upon Sec61 depletion, others were essentially unaffected. As discussed below, this reduction may be due to an inability of ribosomes to stably dock at the membrane in the absence of Sec61 (Kalies et al., 1994). Despite this limitation, the data illustrate that N_exo_ signal anchors do not strictly require Sec61 for insertion, in stark contrast to a signal peptide or N_cyt_ signal anchor. In light of this result, it is noteworthy that N_exo_ signal anchors are the only class of substrates completely resistant to a potent Sec61 inhibitor that prevents opening of the Sec61 channel by signals and N_cyt_ TMDs (McKenna et al., 2017, Morel et al., 2018). Both of these observations can be explained by a model where EMC, not Sec61, plays a primary role during insertion of N_exo_ signal anchors.

### EMC Is Sufficient for N_exo_ Signal Anchor Insertion

To test whether EMC’s insertase function can explain Sec61-independent insertion of N_exo_ signal anchors, we prepared PLs containing purified EMC without or with SRP receptor (SR) and tested their capacity for translocation and membrane insertion (Figure 5B). Importantly, we verified that EMC and EMC/SR PLs are not contaminated with any detectable Sec61 complex (Figure S6D). Consistent with a strict requirement for Sec61 complex, neither prolactin nor ASGR1 showed detectable translocation in EMC or EMC/SR PLs (Figure 5B). By contrast, the β_1_AR-TMD1 was inserted into EMC-containing PLs. Although ∼36% of inserted β_1_AR-TMD1 molecules were in the inverted topology in PLs containing total ER proteins (detected by C-terminal immunoprecipitations; not shown), inverted insertion was very low (<5% of all inserted molecules) in the purified EMC system. Thus, EMC inserted β_1_AR-TMD1 in nearly exclusively the N_exo_ topology.

**Figure S7:**
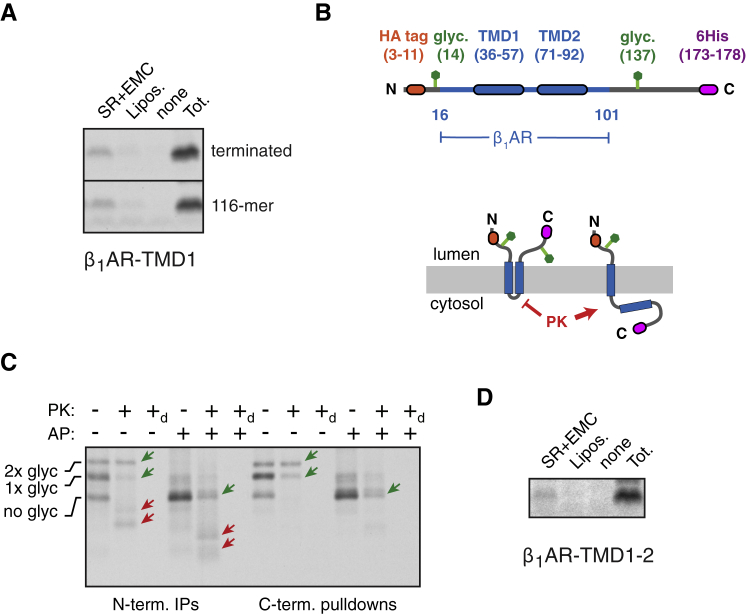
*In Vitro* Topogenesis of a Two-TMD β_1_AR Construct, Related to Figure 5 (A) Insertion assay as in Figure 5B into the indicated proteoliposome preparation. The terminated β_1_AR-TMD1construct (as in Figure 5B) was compared to the same construct stalled at residue 116 (∼60 residues downstream of the TMD, as in Figure S4C). Shown is the immunoprecipitated protease-protected N-terminal fragment diagnostic of successful insertion in the N_exo_ topology. Note that specificity and efficiency of insertion is comparable for the terminated and stalled versions of β_1_AR-TMD1. (B) Diagram of the two-TMD β_1_AR construct (β_1_AR-TMD1-2) and its topology when TMD2 inserts or fails to insert into the membrane. Only the single-spanning form would be accessible to proteinase K (PK) digestion due to the short loop between TMD1 and TMD2. In addition, the double-spanning topology can be glycosylated twice, while the single spanning topology is only glycosylated once. (C) ^35^S-methionine labeled β_1_AR-TMD1-2 was translated in reticulocyte lysate (RRL) in the presence of canine pancreas-derived rough microsomes. Where indicated, the translation reaction contained an acceptor peptide (AP) inhibitor of N-linked glycosylation. The translation products were either left untreated or digested with proteinase K without or with detergent (subscripted d) as indicated. The samples were divided in two and recovered via the N- or C-terminal tag and analyzed by SDS-PAGE and autoradiography. The positions of unglycosylated, singly-glycosylated (1x glyc) or doubly-glycosylated (2x glyc) products are indicated. Green arrows indicate products that are fully protected from protease digestion and represent the double-spanning topology. Red arrows indicate N-terminal protease-protected fragments. Some heterogeneity is observed in the size of these fragments presumably due to heterogeneity in where the protease digests the exposed polypeptide. (D) Insertion assay of β_1_AR-TMD1-2 into the indicated proteoliposome preparations (see Figure 5B). After protease digestion, the N-terminal fragment diagnostic of successful insertion in the N_exo_ topology was recovered and shown in the autoradiograph.

The additional presence of SR stimulated insertion of β_1_AR-TMD1, but not of the tail-anchored protein squalene synthase (SQS). It is likely that this stimulation is due to SR facilitating dissociation of the TMD from SRP. SR alone had no translocation or insertion activity, suggesting that simply delivering β_1_AR-TMD1 to the membrane surface is insufficient to allow insertion. Insertion into EMC/SR PLs was similarly observed for the 116-residue RNCs of β_1_AR-TMD1 (Figure S7A), indicating that the ribosome does not impede EMC-mediated TMD1 insertion. The overall lower insertion efficiencies into these purified EMC PLs relative to PLs containing total ER proteins is probably explained by the lower level of EMC in the purified system (Figure S6D) and the absence of a ribosome docking site normally provided by the Sec61 complex (Kalies et al., 1994). These limitations notwithstanding, we conclude that after targeting, EMC is sufficient to mediate insertion of not only tail-anchored proteins like SQS, but also N_exo_ signal anchors.

### EMC and Sec61 Can Function Sequentially to Insert Two TMDs

The findings thus far indicate that EMC is needed for efficient insertion of TMD1 of GPCRs in the N_exo_ topology, that Sec61 complex is not strictly required for this step, and that purified EMC is sufficient in a reconstituted system to mediate N_exo_ signal anchor insertion. In the context of a full-length GPCR, the next step after TMD1 insertion is TMD2 insertion in the opposite orientation. The reconstitution experiments with ASGR1 indicate that co-translational insertion in this topology requires Sec61 and cannot be mediated by EMC.

To test whether TMD2 of β_1_AR requires Sec61, we analyzed a two-TMD construct (Figure 5C) for insertion in reconstituted PLs containing or lacking the Sec61 complex. Characterization of this construct in native RMs (Figures S7B and S7C) showed that its insertion in the correct double-spanning topology results in a protein that is fully shielded from cytosolic protease due to the inaccessibility of the short intervening cytosolic loop. Polypeptides that fail insertion entirely are digested by cytosolic protease, while those with only the first TMD inserted in the N_exo_ topology generate a protected N-terminal fragment. Insertion in the inverted N_cyt_ topology would result in either a protected C-terminal fragment (single-spanning topology) or a protected internal fragment (double-spanning topology).

Insertion in the correct double-spanning topology was observed in nondepleted PLs, but sharply reduced in Sec61-depleted PLs (Figure 5D). Notably, however, insertion of the first TMD nevertheless occurred in the absence of Sec61, generating the N-terminal protected fragment. Little or no specific protease-protection was observed in reactions containing empty liposomes, or if the PLs were added post-translationally to the reaction (Figure S6E). No clear evidence of inverted insertion products could be seen for this two-TMD construct. Thus, co-translational topogenesis of the first two TMDs of β_1_AR requires Sec61. The point at which Sec61’s role becomes critical is TMD2 insertion, as TMD1 insertion can proceed in its absence. TMD1 insertion can be mediated solely by EMC (Figure S7D), although EMC’s absence is partially tolerated by β_1_AR presumably because its insertion by Sec61 occurs in the correct orientation for a subset of molecules. This indicates that although the correct double-spanning topology can be achieved without EMC, optimal topogenesis requires the combined functions of EMC and Sec61 for insertion of TMD1 and TMD2, respectively.

### Bypass of EMC Dependence by Constraining TMD1 Topology

**Figure 6:**
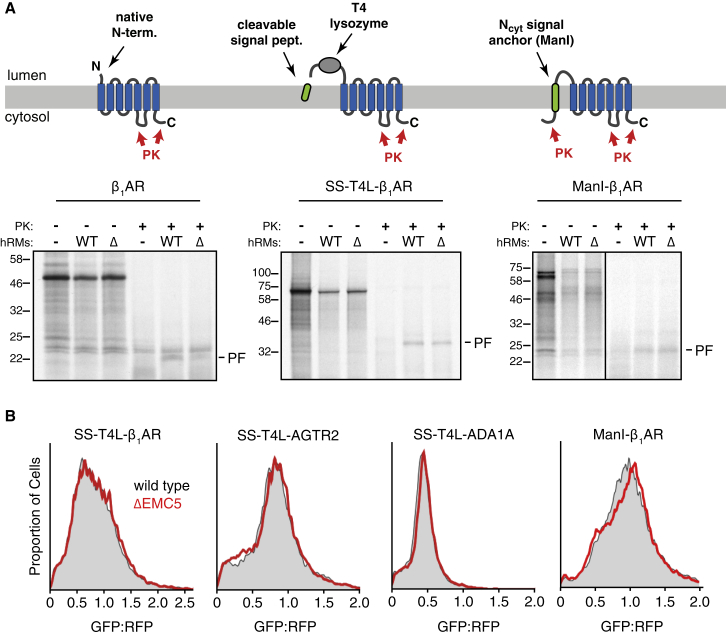
A Sec61-Targeted Signal Sequence or TMD Can Bypass EMC-Dependence *In Vitro* and *In Vivo* (A) Diagram comparing the β_1_AR, SS-T4L-β_1_AR, and ManI-β_1_AR constructs (top) and their analysis of insertion into WT or ΔEMC6 (Δ) hRM as in Figure 2A. PF indicates the protected N-terminal fragment generated by digestion of successfully inserted protein at the loop between TMD5 and TMD6 (see diagrams). (B) Flow cytometry analysis of the indicated constructs in wild-type or ΔEMC5 U2OS cells as in Figure 1. Note that in contrast to the matched constructs lacking the SS-T4L or ManI domains (Figures 1B and 4C), no appreciable consequence of EMC deletion is observed.

The biochemical analyses using simplified N-terminal regions of β_1_AR show that one explanation for the observed requirement for EMC in cells (Figure 1) is its role in topogenesis of TMD1. To investigate whether EMC is required for insertion, folding, or maturation steps beyond TMD1 insertion, we designed versions of β_1_AR whose TMD1 would necessarily insert via Sec61. Sec61 is both necessary and sufficient for signal sequences and N_cyt_ signal anchors to initiate translocation without any appreciable role for EMC. We therefore extended the N terminus of β_1_AR with either a cleavable signal sequence and the secreted protein lysozyme (termed SS-T4L-β_1_AR; see diagram, Figure 6A) or a signal anchor from mannosidase I with a short linker (termed ManI-β_1_AR). Both of these extensions should mediate targeting, initiation of translocation, and commitment of protein topology before TMD1 emerges from the ribosome. Because the polypeptide at this stage would be threaded within the Sec61 channel, TMD1 will enter Sec61 and can insert via its lateral gate in the correct orientation, thereby bypassing EMC’s insertase function.

*In vitro* translocation and protease protection analysis of SS-T4L-β_1_AR and ManI-β_1_AR showed that its insertion occurs similarly in wild-type and ΔEMC6 RMs under conditions where β_1_AR insertion is impaired by more than 50% (Figure 6A). Analysis in cells using the dual-color fluorescent reporter assay showed no difference in either SS-T4L-β_1_AR or ManI-β_1_AR between wild-type and ΔEMC5 cells (Figure 6B). SS-T4L similarly rescued the EMC-dependence of AGTR2 and ADA1A (Figure S6B).

This result has three important implications. First, it strongly argues against any indirect effects of EMC on GPCR levels. Hence, explanations such as globally altered trafficking, degradation, or other general perturbations leading to the reduced GPCR levels (as seen in Figures 1 and 4) seem highly unlikely. Second, the biochemically demonstrated EMC-dependent step of TMD1 insertion characterized *in vitro* must be the mechanistic explanation for reduced GPCR levels in ΔEMC cells observed *in vivo*. Third, the insertase function of EMC used for TMD1 topogenesis appears to be the only step during GPCR biogenesis where EMC is required. Thus, we conclude that EMC’s role in facilitating the biogenesis of many GPCRs is due to its requirement during TMD1 insertion in the N_exo_ topology.

## Discussion

**Figure 7:**
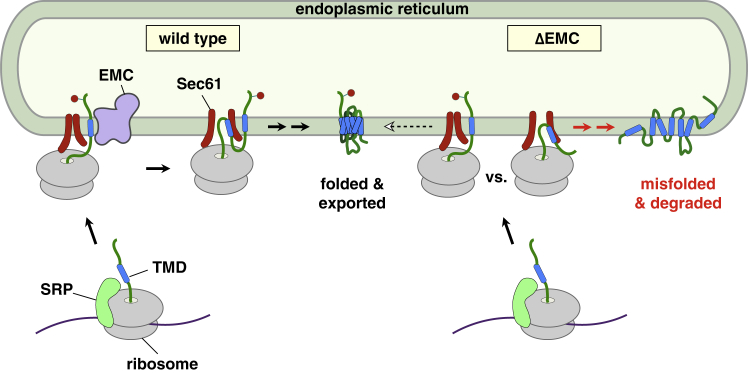
Working Model for the Roles of EMC and Sec61 Complex in GPCR Biogenesis The left half of the diagram shows the normal situation (wild-type), and the right half depicts the consequence of EMC deletion (ΔEMC). After targeting via SRP, the N_exo_ signal anchor is inserted via EMC, likely near the Sec61 complex to which the ribosome is probably docked. Downstream TMDs are inserted by Sec61. In the absence of EMC, the N_exo_ signal anchor of most of the nascent chains fails to insert in the correct topology, resulting in a misfolded and degraded protein. Depending on the substrate, some nascent chains are inserted appropriately by the Sec61 complex even in the absence of EMC, leading to a small population of correctly folded final protein.

We propose the following working model for the role of EMC in GPCR topogenesis (Figure 7). A nascent signal anchor will be recognized by SRP (Figure 3B) and targeted to the ER membrane, where the ribosome will dock onto the Sec61 complex. Next, the signal anchor will dissociate from SRP in close proximity to both the membrane and Sec61 complex. At this stage, features of the signal anchor and flanking regions will determine the extent to which it requires EMC for insertion in the N_exo_ orientation (Figures 4D, 4E, and S5A). Increased length, moderate hydrophobicity, and ambiguous flanking charge distribution all contribute to EMC dependence. TMDs with these features apparently cannot effectively engage Sec61 in the N_exo_ orientation, resulting in inverted (e.g., Figure 3A) or failed insertion when EMC is absent. After the first TMD is correctly inserted, the topologic ‘reading frame’ is set, and the remaining TMDs are inserted by a process that does not need EMC (Figure 6) but does require Sec61 (Figure 5D). EMC therefore plays a critical role in initiating the accurate topogenesis of many GPCRs. We find that even EMC-independent N_exo_ signal anchors (e.g., from LepB) can use purified EMC for insertion (unpublished results), explaining why its insertion is unaffected by Sec61 depletion (Figure S6B). Thus, EMC is a major eukaryotic insertase for N_exo_ signal anchors (this study) and tail-anchored membrane proteins (Guna et al., 2018).

We favor a mechanism by which N_exo_ TMDs are inserted by EMC in proximity to the Sec61 translocon (Figure 7). Proximity to Sec61 is posited because this is the site of ribosome docking at the ER (Kalies et al., 1994, Voorhees et al., 2014) and Sec61 is known to be near nascent N_exo_ signal anchors (Heinrich et al., 2000, High et al., 1993). Despite its proximity, Sec61 is apparently dispensable for N_exo_ signal anchor insertion (Figure 5A). The partial N_exo_ insertion defect seen in the absence of Sec61 can be explained by its role in docking and orienting the ribosome at the membrane (Kalies et al., 1994). Consistent with this interpretation, a potent Sec61 inhibitor that is permissive for ribosome binding shows no discernible effect on N_exo_ signal anchor insertion despite strongly inhibiting N_cyt_ substrates (McKenna et al., 2017, Morel et al., 2018). Thus, N_exo_ TMD insertion is likely to be mediated by EMC, although we cannot know whether the TMD first attempted to engage Sec61 or used EMC directly.

N_exo_ signal anchor insertion by EMC means that N_exo_ and N_cyt_ hydrophobic elements use different mechanisms of membrane insertion. Signal sequences and N_cyt_ signal anchors use Sec61’s lateral gate (Li et al., 2016, Voorhees and Hegde, 2016), which must necessarily open to allow insertion. Hence, these substrates strictly require Sec61 (Figure 5A) (Görlich and Rapoport, 1993), cannot use EMC (Figure 5B), and are unaffected by EMC deletion in cells (e.g., Figure 1). By contrast, N_exo_ signal anchor insertion can be mediated by EMC (Figure 5B) and proceeds well when Sec61 is depleted (Figures 5A, S6B, and S6C). The most attractive mechanism to explain these observations is a “sliding” model (Cymer et al., 2015) where the N_exo_ signal anchor inserts headfirst via EMC near the outside surface of Sec61’s lateral gate. In the absence of EMC, some TMDs might still be able to insert with reasonable efficiency in the N_exo_ orientation by sliding into the more limited protein-lipid interface at Sec61’s lateral gate as previously speculated (Cymer et al., 2015). TMD features favorable for this EMC-independent reaction appear to be a short length and high hydrophobicity. The most extensively studied N_exo_ model protein (LepB) meets these criteria and can insert into liposomes containing only Sec61 (Heinrich et al., 2000). LepB insertion solely by Sec61 was thought to apply to all N_exo_ signal anchor insertion, an assumption that appears to have been premature. The fact that N_exo_ signal anchors of multi-pass membrane proteins typically have critical roles in that protein’s folding or function probably constrains their ability to evolve into signal anchors that can efficiently insert using only the Sec61 complex. Analogous constraints for N_cyt_ signal anchors may similarly warrant the need for other Sec61-associated factors for efficient insertion, an idea that remains to be explored in detail.

While N_exo_ signal anchors would not obligately use Sec61 for insertion, they can probably engage Sec61 at its lateral gate after insertion. This idea is favored by Sec61’s close proximity to the nascent chain via ribosome binding (Kalies et al., 1994), the signal-binding capacity of Sec61’s lateral gate (Li et al., 2016, Voorhees and Hegde, 2016), and the observed Sec61-TMD crosslinking (High et al., 1993). The signal anchor would then be positioned ideally for interacting with the next TMD, whose insertion would occur via Sec61 (Figure 5D). Interactions between TMDs are thought to be an important, but poorly understood aspect of multi-pass membrane proteins (Heinrich and Rapoport, 2003, Ismail et al., 2006, Meacock et al., 2002, Skach and Lingappa, 1993).

Both N_exo_ signal anchors and tail-anchored proteins contain relatively short unstructured translocated domains (Figures S5B and S5C). While EMC can insert these two classes of proteins, it apparently cannot translocate large soluble domains using either a signal sequence or N_cyt_ signal anchor (Figure 5B). This limitation might indicate that unlike the Sec complex (Li et al., 2016, Voorhees and Hegde, 2016), EMC cannot simultaneously accommodate a hydrophobic domain and the soluble translocating polypeptide that follows it. EMC may therefore be analogous to how the prokaryotic insertase YidC (Samuelson et al., 2000), possibly a distant homolog of EMC3 (Anghel et al., 2017), contains a route into the membrane interior but not across the lipid bilayer (Kumazaki et al., 2014). Like current models of YidC (Dalbey et al., 2014), EMC can function sequentially with the Sec complex to successively insert two TMDs. Our *in vitro* reconstitution of the biogenesis of multi-pass membrane proteins that depend on both EMC and the Sec61 complex now paves the way for mechanistic and structural dissection of how they might cooperate during this poorly understood process.

The capacity to insert EMC substrates at least partially by other routes would explain why EMC is non-essential at the cellular level (Guna et al., 2018, Jonikas et al., 2009), but causes ER stress due to an increase of mis-inserted products. The greater demand for accurate levels of membrane proteins during intercellular interactions, signaling, and *trans*-bilayer transport may explain why EMC shows much stronger phenotypes in multicellular contexts (Richard et al., 2013, Satoh et al., 2015). Indeed, GPCRs have exceptionally broad physiologic roles in metazoans, but their precise levels are less critical at the single-cell level. The possibility that EMC activity might be selectively modulated to affect topogenesis, and hence function, of key GPCRs as a means of cellular regulation warrants future study.

Several earlier studies have shown that membrane proteins, many of which contain multiple TMDs, are preferentially impacted by knockout of EMC (Louie et al., 2012, Richard et al., 2013, Satoh et al., 2015, Shurtleff et al., 2018). In one of these studies, proximity labeling of ribosomes near EMC in yeast showed some enrichment for ribosomes synthesizing membrane proteins that might represent direct EMC clients (Shurtleff et al., 2018). The observation that many of these candidates do not have N_exo_ signal anchors raises the intriguing possibility that they rely on EMC in a non-insertase role or use EMC’s insertase activity for downstream TMDs. Experimental support for these ideas is currently lacking because it is not known whether any of these yeast candidates implicated by proximity ribosome labeling are affected in their biogenesis in EMC knockouts. Conversely, it is unclear which of the proteins that decrease in acute EMC knockdowns in human cells (Shurtleff et al., 2018) are adjacent to EMC during biogenesis. Thus, defining the proteins and specific biosynthetic events that directly rely on EMC remains an important future goal.

## STAR★Methods

### Key Resources Table

**Table:** 

REAGENT or RESOURCE	SOURCE	IDENTIFIER
**Antibodies**		

Rabbit polyclonal Calnexin	Enzo Lifesciences	Cat#ADI-SPA-865; RRID: AB_10618434
Rabbit polyclonal Sec61α	Song et al., 2000	N/A
Rabbit polyclonal Sec61β	Fons et al., 2003	N/A
Rabbit polyclonal Sec62	Garrison et al., 2005	N/A
Rabbit polyclonal Sec63	Garrison et al., 2005	N/A
Rabbit polyclonal SP12	Görlich and Rapoport, 1993	N/A
Rabbit polyclonal SRP receptor	Görlich and Rapoport, 1993	N/A
Rabbit polyclonal TRAM	Fons et al., 2003	N/A
Rabbit polyclonal TRAPα	Fons et al., 2003	N/A
Rabbit polyclonal EMC2	Proteintech	Cat#25443-1-AP; RRID: AB_2750836
Rabbit polyclonal EMC4	Abcam	Cat#Ab123719; RRID: AB_10951091
Rabbit polyclonal EMC5	Abcam	Cat#Ab174366; RRID: AB_2750837
Rabbit polyclonal EMC6	Abcam	Cat#Ab84902; RRID: AB_1925516
Rabbit polyclonal SRP54	BD Biosciences	Cat#610941; RRID: AB_398254
Rabbit polyclonal RPL8 (uL2)	Abcam	Cat#Ab169538; RRID: AB_2714187
Rabbit monoclonal RPS24 (eS24)	Abcam	Cat#Ab196652; RRID: AB_2714188

**Chemicals, Peptides, and Recombinant Proteins**		

Ni-NTA agarose	QIAGEN	Cat#30210
Protein A Resin	Repligen	Cat#CA-PRI-0100
Bismaleimidohexane (BMH)	Thermo	Cat#22330
EasyTag L-[^35^S]-Methionine	Perkin Elmer	Cat#NEG709A005MC
CAP (diguanosine triphosphate cap)	New England Biolabs	Cat#S1404L
RNasin	Promega	Cat#N251
Amino acid kit	Sigma-Aldrich	Cat#09416
SP6 Polymerase	New England Biolabs	Cat#M0207L
Creatine kinase	Roche	Cat#127566
Creatine phosphate	Roche	Cat#621714
Cycloheximide	Sigma-Aldrich	Cat#C4859; CAS: 66-81-9

**Experimental Models: Cell Lines**		

Flp-In 293 T-Rex Cells WT	Guna et al., 2018	N/A
Flp-In 293 T-Rex Cells ΔEMC6	Guna et al., 2018	N/A
U2OS Flp-In Cells WT	Guna et al., 2018	N/A
U2OS Flp-In Cells ΔEMC5	Guna et al., 2018	N/A
U2OS Flp-In Cells ΔEMC5+EMC5 Rescue	Guna et al., 2018	N/A

**Bacteria and Virus Strains**		

*E. coli* BL21(DE3) pLysS	Thermo Fisher	Cat#C606003

**Recombinant DNA**		

SP64 HA-β_1_AR-β-6His	This study	N/A
SP64 HA-β_1_AR (TM1)-β-6His	This study	N/A
SP64 HA-β_1_AR (TM1-2)-β-6His	This study	N/A
SP64 HA-β_1_AR (TM1-3)-β-6His	This study	N/A
SP64 HA-β_1_AR (TM1-4)-β-6His	This study	N/A
SP64 HA-β_1_AR (TM1-5)-β-6His	This study	N/A
SP64 HA-β_1_AR (TM1-6)-β-6His	This study	N/A
SP64 SS-HA-T4L-β_1_AR-β-6His	This study	N/A
SP64 HA-β_1_AR(ΔCL3)-β-6His	This study	N/A
SP64 HA-β_1_AR(ΔTM3)-β-6His	This study	N/A
SP64 HA-V1BR(TM1)-β-6His	This study	N/A
SP64 HA-VN1R5(TM1)-β-6His	This study	N/A
SP64 HA-MTR1L(TM1)-β-6His	This study	N/A
SP64 HA-OR3A2(TM1)-β-6His	This study	N/A
SP64 HA-OR2L5(TM1)-β-6His	This study	N/A
SP64 HA-ACM1(TM1)-β-6His	This study	N/A
SP64 HA-O52N2(TM1)-β-6His	This study	N/A
SP64 HA-ACTHR(TM1)-β-6His	This study	N/A
SP64 HA-hADRB1(TM1)-β-6His	This study	N/A
SP64 HA-CNR2(TM1)-β-6His	This study	N/A
SP64 HA-O56A3(TM1)-β-6His	This study	N/A
SP64 HA-NK1R(TM1)-β-6His	This study	N/A
SP64 HA-CCKAR(TM1)-β-6His	This study	N/A
SP64 HA-TAAR5(TM1)-β-6His	This study	N/A
SP64 HA-ADA1A(TM1)-β-6His	This study	N/A
SP64 HA-AGTR2(TM1)-β-6His	This study	N/A
SP64 Nat-CNR2(TM1)-β-6His	This study	N/A
SP64 Nat-ACM1(TM1)-β-6His	This study	N/A
SP64 Nat-V1BR(TM1)-β-6His	This study	N/A
pcDNA3.1 GFP-P2A-RFP	Itakura et al., 2016	N/A
pcDNA3.1 HA-β_1_AR-GFP-P2A-RFP	This study	N/A
pcDNA5 GFP-P2A-RFP-SQS	This study	N/A
pcDNA5 GFP-P2A-RFP-ASGR1	This study	N/A
pcDNA5 AGTR2 GFP-P2A-RFP	This study	N/A
pcDNA5 CNR2 GFP-P2A-RFP	This study	N/A
pcDNA5 ADA1A GFP-P2A-RFP	This study	N/A
pcDNA3.1 SS-HA-T4L-β_1_AR-GFP-P2A-RFP	This study	N/A
pcDNA5 SS-HA-T4L-CNR2-GFP-P2A-RFP	This study	N/A
pcDNA5 SS-HA-T4L-AGTR2-GFP-P2A-RFP	This study	N/A
pcDNA5 SS-HA-T4L-ADA1A-GFP-P2A-RFP	This study	N/A
SP64 Bovine Prolactin	Fons et al., 2003	N/A
SP64 Hamster PrP	Fons et al., 2003	N/A
SP64 ASGR1-3F4	This study	N/A
SP64 3xHA-LepB	This study	N/A
SP64 HA-β_1_AR (TM1 1A)-β-6His	This study	N/A
SP64 HA-β_1_AR (TM1 2A)-β-6His	This study	N/A
SP64 HA-β_1_AR (TM1 Δ1)-β-6His	This study	N/A
SP64 HA-β_1_AR (TM1 Δ2)-β-6His	This study	N/A
SP64 HA-β_1_AR (TM1 Δ3)-β-6His	This study	N/A
SP64 HA-β_1_AR (TM1 Δ4)-β-6His	This study	N/A
SP64 HA-β_1_AR (TM1 −3)-β-6His	This study	N/A
SP64 HA-β_1_AR (TM1 0)-β-6His	This study	N/A
SP64 HA-β_1_AR (TM1 +1)-β-6His	This study	N/A
SP64 HA-β_1_AR (TM1 +2)-β-6His	This study	N/A
SP6 HA-β_1_AR (TM1 +4)-β-6His	gBlock (IDT)	N/A
SP64 HA-β_1_AR (TM1 +5)-β-6His	This study	N/A
SP64 HA-β_1_AR (TM1 +6)-β-6His	This study	N/A
SP6 HA-β_1_AR (TM1 1L)-β-6His	gBlock (IDT)	N/A
SP6 HA-β_1_AR (TM1 2L)-β-6His	gBlock (IDT)	N/A
SP6 HA-β_1_AR (TM1 3L)-β-6His	gBlock (IDT)	N/A
pcDNA5 TRAM2-GFP-P2A-RFP	This Study	N/A
SP64-TRAM2	This Study	N/A
SP64-HA-MAN1A1-β_1_AR-β-6His	This Study	N/A
pcDNA5 HA-MAN1A1-β_1_AR-GFP-P2A-RFP	This Study	N/A
SP6 HA-LEP (TM1)-β-6His	gBlock (IDT)	N/A
SP6 HA-LEP-Ext (TM1)-β-6His	gBlock (IDT)	N/A
SP64 HA-β_1_AR-β-6His	This study	N/A
SP64 HA-β_1_AR (TM1)-β-6His	This study	N/A

**Software and Algorithms**		

FlowJo	FlowJo	https://www.flowjo.com/
Adobe Illustrator	Adobe	https://www.adobe.com/uk/creativecloud.html
UniProt	UniProt	https://www.uniprot.org/

**Other**		

SuperSignal West Pico Chemiluminescent substrate	Thermo Fisher	Cat#34080
Rabbit Reticulocyte Lysate Mix	Sharma et al., 2010	N/A
DMEM, high glucose, GlutaMAX, pyruvate	Thermo Fisher	Cat#10569010
Tetracycline-free Fetal Calf Serum (FCS)	BioSera	Cat#FB-1001T/500
PonceauS Solution	Sigma-Aldrich	Cat#P-7170-1L
TransIT 293	Mirus	Cat#MIR 2705

### Contact for Reagent and Resource Sharing

Further information and requests for resources and reagents should be directed to and will be fulfilled by the Lead Contact, Ramanujan S. Hegde (rhegde@mrc-lmb.cam.ac.uk).

### Experimental Model and Subject Details

#### Cell lines

All cell lines were cultured in Dulbecco’s Modified Eagle’s Medium (DMEM) with 10% fetal calf serum (FCS). In cases where the cells contained a stably expressed doxycycline-inducible reporter, tetracycline-free FCS was used as well as 15 μg/ml blasticidin and 100 μg/ml hygromycin. All cell lines used in this study (listed in the Key Resources Table) have been described and characterized previously (Guna et al., 2018). They include the following: Flp-In 293 T-Rex cells (wild-type and ΔEMC6), and U2OS Flp-In cells (WT, ΔEMC5, ΔEMC5+EMC5 rescue). Cell lines were routinely validated for the presence or disruption of the indicated EMC subunit (by immunoblotting) and for the presence of an insert at the Frt locus (by antibiotic resistance markers and immunoblotting of doxycycline-induced cells). All cell lines are female. They were not authenticated further.

### Method Details

#### Constructs

The parent β_1_AR construct for *in vitro* translation was created by inserting the coding region of residues 20-424 of turkey β_1_AR-B6m23 (Warne et al., 2009) into an SP64 based vector containing an HA affinity tag at the N terminus and the unstructured cytosolic domain of Sec61β (residues 2-69, with the single Cysteine and predicted Glycosylation acceptor sequence mutated to Serine and Glutamine, respectively) followed by a 6-Histidine tag at the C terminus. A glycosylation acceptor site (NGT) was introduced at residues 22-24 within the β_1_AR sequence. From this parent construct, versions lacking the HA tag, glycosylation site, cytosolic loop 3 (CL3 residues 233-262), and TMD3 (residues 109-148) were generated by standard subcloning methods. β_1_AR-TMD1 was created by deleting everything downstream of the beginning of TMD2 from the parent cassette. Similar approaches were used to create constructs β_1_AR-TMD1-2 through β_1_AR-TMD1-6. All GPCR-TMD1, β_1_AR TMD1 mutants, and LEP TMD mutant constructs (Figures 4 and S5A) were made by replacing the β_1_AR TMD1 with the respective first TMDs of indicated GPCRs, or mutants of either β_1_AR TMD1 or LEP TMD1, including up to 15aa of the N-terminal native sequence (or the entire native N terminus where indicated) and the entire cytosolic loop 2 (CL2) sequence preceding TMD2. Any native cysteines were mutated to serine. Methionines were added where necessary by mutating hydrophobic residues to allow for efficient detection by autoradiography. The coding sequences for human ASGR1 (Görlich and Rapoport, 1993) and bacterial leader peptidase (LEP) with the TMD2 removed (Heinrich et al., 2000), were placed into an SP64 based vector containing a 3F4 epitope at the C terminus or 3xHA and glycosylation tag at the N terminus, respectively. For the construction of the *in vivo* β_1_AR fluorescent reporter, the sequence encoding HA-β_1_AR was sub-cloned into a pcDNA3.1 based vector containing a C-Terminal GFP-P2A-RFP reporter (Itakura et al., 2016). For all other fluorescent reporters, a parent cassette was first created by sub-cloning the GFP-P2A-RFP fluorescent reporter into a pcDNA5/FRT/TO vector backbone. The coding sequences of CNR2 (NP_001832.1), AGTR2 (NP_000677.2), and ADA1A (NP_000671.2) were then inserted into this parent cassette with the GFP-P2A-RFP reporter at the C terminus. The coding regions for both ASGR and SQS (Guna et al., 2018) were inserted at the 3′ end of the GFP-P2A-RFP reporter within the pcDNA5 cassette. A gene block (IDT) encoding the signal sequence of prolactin followed by an HA-epitope tag and the sequence for full-length Phage T4 Lysozyme was appended to all GPCR-GFP-P2A-RFP cassettes using Gibson Assembly (NEB). The T4 Lysozyme sequence (residues 2-161) had all native cysteines and predicted glycosylation acceptor sites mutated to serine or glutamine, respectively). Additionally, the N-terminal HA-epitope tag preceding the GPCR sequence was removed and replaced by the appended SS-HA-T4L sequence. SS-T4L-β_1_AR for *in vitro* expression in an SP64 based cassette was cloned in a similar manner. A gene block (IDT) encoding an HA tag and the TMD of MAN1A1 (NP_005898.2), including native N and C-terminal flanking residues (aa 33-75), was appended to the N terminus of β_1_AR in both the SP64 cassette and the pcDNA5 GFP-P2A-RFP cassette using Gibson Assembly. As indicated in the Key Resources Table, several β_1_AR TM1 constructs and LEP TM1 constructs were ordered as gBlocks containing the SP6 promoter and coding sequence of interest. PCR for subsequent *in vitro* transcription was carried out directly from these gBlocks. TRAM2 was PCR amplified from a human cDNA library and then inserted into the SP64 cassette using restriction cloning. Subsequently, the coding sequence of the TRAM2 mRNA was PCR amplified and inserted a parent pcDNA5-GFP-P2A-RFP cassette by Gibson Assembly.

#### Flow cytometry analysis

Analysis of reporter expression by flow cytometry was similar to previously described methods (Guna et al., 2018, Itakura et al., 2016) as follows. Transient transfection of fluorescent reporter constructs was performed using either Mirus TransIT 293 (for HEK293 T-Rex cells) or Mirus TransIT 2020 (for U2OS cells) according to manufacturer’s instructions. In all experiments, 1μg/ml of total plasmid was transfected into a dish containing complete medium. The amount of the fluorescent reporter plasmid was titrated individually for each protein of interest based on transfection efficiency and expression levels, and a non-expressing plasmid was used to maintain equal amounts of total plasmid transfected (1μg/ml). For the U2OS ΔEMC5 rescue cells, re-expression of EMC5 was induced for 24-30 hours with 1 μg/ml of doxycycline prior to reporter plasmid transfection. Following transfection, cells were trypsinized, washed once with PBS and pelleted at room temperature at 500 x g for 5 min. The cells were resuspended in 500μl PBS, passed through a 70 μm filter, and analyzed by flow cytometry using a Beckton Dickinson LSR II instrument. 20,000 GFP positive cells (or RFP for SQS and ASGR1) were selected for analysis of GFP and RFP fluorescence. Then cells were further gated for moderate expression levels using the fluorescent protein (FP) that reports on translation (not the FP appended to the protein of interest). Data analysis was performed using FlowJo software.

#### In vitro transcription and translation

*In vitro* transcription was performed with SP6 polymerase using PCR products as the template (Sharma et al., 2010) as follows. The transcription reactions were conducted with 5-20 ng/μl PCR product in 40 mM HEPES pH 7.4, 6 mM MgCl_2_, 20 mM spermidine, 10 mM reduced glutathione, 0.5 mM ATP, 0.5 mM UTP, 0.5 mM CTP, 0.1 mM GTP, 0.5 mM CAP, 0.4-0.8 U/μl RNasin and 0.4 U/μl SP6 polymerase at 37°C. *In vitro* translation in RRL was as described previously in detail (Feng and Shao, 2018, Sharma et al., 2010). In brief, translations were for 20-45 minutes at 32°C unless indicated otherwise in the individual figure legends. Translation reactions typically contained 33% by volume nuclease-treated RRL, 0.5 μCi/μl ^35^S-methionine, 20 mM HEPES, 10 mM KOH, 40 μg/ml creatine kinase, 20 μg/ml pig liver tRNA, 12 mM creatine phosphate, 1 mM ATP, 1 mM GTP, 50 mM KOAc, 2 mM MgCl_2_, 1 mM reduced glutathione, 0.3 mM spermidine and 40 μM of each amino acid except methionine. The transcription reaction was added to 5% by volume to the translation reaction without further purification. For translation reactions in the presence of human cell-derived rough microsomes (hRMs), 0.25-1 μL of hRMs (at concentration that gives an absorbance at 280 nm of 75) were added to a 10μl translation reaction. Each batch of hRMs was titrated in preliminary experiments to achieve equal translation levels, allowing for functional comparisons between various microsomes.

#### Preparation of rough microsomes

Canine pancreas-derived rough microsomes (cRM) were prepared by minor modifications of a previous protocol (Walter and Blobel, 1983). In brief, freshly harvested canine pancreas was manually dissected at 4°C to remove blood vessels and connective tissue, then minced with a razor blade. 4 mL of ice-cold homogenization buffer (50 mM HEPES, pH 7.4, 50 mM KOAc, 6 mM Mg(OAc)_2_, 1 mM EDTA, 250 mM sucrose, 1 mM DTT) was added per gram of tissue, and supplemented with one crushed tablet of EDTA-free “Complete” protease inhibitor (Roche) per 50 mL total volume. All subsequent procedures were carried out at 4°C. The mixture was homogenized by 5 passes up and down with a motorized Potter-Elvehjem homogenizer and centrifuged for 10 min at 1,000 x g in a JA-17 rotor. The supernatant was recovered and centrifuged a second time at 10,000 x g in a JA-17 rotor. Aliquots of the supernatant from this second spin were pooled, then transferred to ultracentrifuge tubes. The samples were under-layered with one-third the volume of a sucrose cushion (1.3 M sucrose, 50 mM HEPES, pH 7.4, 50 mM KOAc, 6 mM Mg(OAc)_2_, 1 mM EDTA, 1 mM DTT) and centrifuged for 2.5 h at 140,000 x g (40,000 rpm) in the Ti50.2 rotor (Beckman). The supernatant was removed by aspiration, and the pellet was resuspended by manual homogenization in a dounce using 1 mL resuspension buffer (250 mM sucrose, 50 mM HEPES, pH 7.4, 1 mM DTT) per gram of starting tissue. The preparation was finally adjusted to an absorbance of 50 when measured at 280 nm in 1% SDS. The microsomes were frozen in liquid nitrogen and stored at −80°C. Preparation of microsomes from HEK293-based cells was slightly modified from earlier protocols (Zhang et al., 2013). Briefly, ten 15 cm plates of Flp-In 293 T-Rex cells (wild-type or ΔEMC6) were grown to 80%–100% confluency, collected in ice-cold PBS, sedimented at 500 x g for 5 min at 4°C, and washed twice in ice-cold PBS. The cell pellet was resuspended in 3 pellet volumes of ice-cold sucrose buffer (10mM HEPES, pH 7.4, 250 mM sucrose, 2 mM MgCl_2_). Cells were lysed in the cold (4°C) by ∼25-30 passes through a 26 guage needle using a 1 mL syringe. The lysates were clarified of nuclei and debris by centrifugation twice at 3,800 x g for 30 min at 4°C in a tabletop micro-centrifuge. The supernatant was centrifuged at 75,000 x g for 1 hr at 4°C in an MLA-80 rotor (Beckman Coulter). The supernatant was discarded and the resulting membrane pellet was resuspended in microsome buffer (10 mM HEPES, pH 7.4, 250 mM sucrose, 1 mM MgCl_2_, 0.5 mM DTT). Total microsome resuspension volume was adjusted such that the absorbance at 280nm was 75 when measured in 1% SDS.

#### Protease protection assays

Immediately following the translation reaction, the samples were placed on ice and 10% of the reactions were set aside for analysis by SDS-PAGE and autoradiography of total products. The remainder was subjected to protease digestion by the addition of proteinase K (PK) to a final concentration of 0.5 mg/ml and incubated on ice for 50 min. To stop the digestion reaction, PMSF was added to 5 mM, incubated on ice for 2-5 min, and the entire reaction transferred to 10 volumes of boiling 1% SDS, 100 mM Tris-Cl, pH 8.0. For subsequent immunoprecipitations and pull-downs, samples were diluted 10-fold in ice-cold immunoprecipitation buffer (1x PBS supplemented with an additional 250 mM NaCl, 0.5% TX-100, 10 mM imidazole). Subsequently, samples were added to 10μl (packed) of either Nickel-NTA resin (to capture 6His-tagged proteins), or Protein A agarose plus the appropriate antibody typically used at 1:300 dilution. Immunoprecipitations were incubated for 2 hours rotating at 4°C. Following binding, the resin was washed twice with 50-100 resin volumes of immunoprecipitation buffer, eluted with sample buffer, and analyzed directly by SDS-PAGE and autoradiography.

#### Carbonate extraction

Translation reactions were chilled on ice, layered on a sucrose cushion [20% w/v sucrose in physiological salt buffer (PSB): 100 mM KOAc, 50 mM HEPES pH 7.4, 2 mM Mg(OAc)_2_], and centrifuged at 186,000 x g for 20 min. The membrane pellet was resuspended in 20 μl PSB, 10% was set aside as the total membrane fraction, and the remainder was diluted 100-fold in 100 mM Na_2_CO_3_ pH 11.5 and incubated on ice for 25 min. The resulting Na_2_CO_3_ extracted membranes were isolated through centrifugation in the TLA120.2 rotor (Beckman Coulter) at 70,000 rpm at 4°C for 30min. The Na_2_CO_3_ extracted pellet was resuspended in SDS-PAGE sample buffer. After SDS-PAGE, the gels were either exposed to detect translation products by autoradiography, or subjected to immunoblotting to assess the separation of endogenous membrane and lumenal proteins (α-Calnexin 1:5,000 or α-PDI 1:1,000).

#### Analysis of ribosome-nascent chain complexes

For generating templates of truncated mRNAs, PCR was used to amplify the desired region using a 5′ primer that anneals slightly upstream of the SP6 promoter and a 3′ primer that anneals at the desired site of truncation. The 3′ primer additionally encodes the residues “MLKV” to improve radiolabeling (via the methionine) and stability of the peptidyl-tRNA from hydrolysis during gel electrophoresis (Shao et al., 2013). The PCR products were used in transcription and translation reactions as described above to generate ribosome-nascent chain complexes (RNCs). Following translation, cycloheximide was added to a final concentration of 50 μg/ml prior to the addition of membranes. Microsomes were then added as indicated in the figure legends, incubated for 32°C for 15 min, then returned to ice for subsequent protease-protection assays as described above.

#### Cysteine crosslinking of integration intermediates

Cysteine crosslinking reactions started with 40 μl RNC translation reactions as described above. An aliquot of the reaction was analyzed directly to visualize the total translation products. The remainder was centrifuged at 55,000 rpm in the TLA-55 rotor (Beckman Coulter) for 20 min at 4°C through a 20% sucrose cushion (in PSB) to isolate membranes. The resulting microsome pellet was resuspended in 20 μl of PSB, and the sulfhydryl-reactive crosslinker bismaleimidohexane (BMH) was added to a final concentration of 250 μM, then incubated on ice for 30 min. The crosslinking reaction was quenched by the addition of an equal volume of quenching buffer (40 mM Tris-Cl, pH 7.4, 20 mM EDTA, and 10 mM β-Mercaptoethanol), then digested with 0.15 mg/ml RNase A on ice for 30 min, and denatured in SDS-PAGE sample buffer. Products were immunoprecipitated using an antibody against Sec61β antibody (1:300) that only recognizes the endogenous protein containing its native N terminus (and not the Sec61β region in our constructs).

#### Purification of EMC and SRP receptor

SRP receptor (SR) was purified using an affinity resin coupled to anti-SR-alpha as described (Görlich and Rapoport, 1993). In brief, 30 mL of dog pancreatic rough microsomes were adjusted to a final concentration of 0.4% digitonin. The mixture was centrifuged for 40 min at 100,000 rpm in a TLA110 rotor. The supernatant was removed, and the pellet was resuspended in extraction buffer (20 mM HEPES, pH 7.4, 400 mM KOAc, 12 mM Mg(OAc)_2_, and 3% digitonin). After 10 min on ice, the mixture was centrifuged for 60 min at 100,000 rpm in a TLA110 rotor. The supernatant was applied at 4°C at 10 ml/hr to a 2.5 mL column that contained 2 mg/ml affinity-purified antibodies raised against a peptide (corresponding to residues 137-150) of the alpha subunit of the canine SRP receptor. The column was washed with 50 mL of equilibration buffer (50 mM HEPES, pH 7.8, 500 mM KOAc, 5 mM 2-mercaptoethanol, 15% w/v glycerol, and 0.5% digitonin). Elution of the SRP receptor was carried out at room temperature at a flow rate of 2 ml/hr with 1 mg/ml of the peptide against which the antibodies were raised in 50 mM HEPES, pH 7.8, 750 mM KOAc, 5 mM Mg(OAC)_2_, 0.5 mM GTP, 15% glycerol, and 0.5% digitonin. The elution was diluted 5-fold, bound to a 0.5 mL S-Sepharose column and washed twice with 5 mL of 50 mM HEPES, pH 7.8, 150 mM KOAc, 5 mM Mg(OAC)_2_, 15% glycerol, and 0.3% deoxy-BigChap. SR was then eluted with 50 mM HEPES, pH 7.4, 750 mM KOAc, 5 mM Mg(OAc)_2_, 0.3% deoxy-BigChap (DBC). EMC was purified as described previously (Guna et al., 2018) and minor contaminants removed by a cation exchange step as follows. Flp-In 293T-Rex cells with stably expressed EMC5-FLAG were induced by the addition of 1μg/mL of doxycycline for 48 hr prior to collection. A ∼2.5 g pellet of cells was resuspended in 20 mL of solubilization buffer [50 mM HEPES, 200 mM NaCl, 2 mM Mg(OAc)_2_, 1% DBC, and EDTA free Protease Inhibitor cocktail (Roche)]. After 30 min on ice, the lysate was cleared by centrifugation at 21,000 x g for 20 min at 4°C in the JA-25.50 rotor (Beckman Coulter). The cleared lysate was then added to 500 μl (packed) of anti-FLAG M2 affinity gel pre-equilibrated in wash buffer 1 [50 mM HEPES, 200 mM NaCl, 2 mM Mg(OAc)_2_, 0.3% DBC] and incubated at 4°C rotating for 1 hr. The affinity resin was collected by brief centrifugation and washed 5 times in 8 resin volumes of wash buffer 1. EMC was eluted in 1 mL elution buffer [50 mM HEPES, 100 mM NaCl, 2 mM Mg(OAc)_2_, 0.3% DBC, and 250 μg/mL 3xFLAG peptide] by rotating for 30 min at room temperature. The eluate was then passed through a gravity flow column containing 150 μl (packed) SP-Sepharose Fast-Flow that was pre-equilibrated in wash buffer 2 [50 mM HEPES, 50 mM NaCl 2 mM Mg(OAc)_2_, 0.3% DBC]. The column was washed 4 times with 10 resin volumes of wash buffer 2, and eluted in 200 μl of 50 mM HEPES, 200 mM NaCl, 2 mM Mg(OAc)_2_, and 0.25% DBC.

#### Preparation of Total and Sec Depleted protein extracts

1 mL of canine rough microsomes (at an absorbance at 280 nm of 50) was diluted in an equal volume of ice-cold 50 mM HEPES, pH 7.4, 250 mM Sucrose, 0.15% DBC. Membranes were collected by centrifugation at 100,000 rpm for 15 min at 4°C in the TL100.3 rotor (Beckman Coulter), resuspended in 1 mL of 400 mM KOAc, 50 mM HEPES, 5 mM Mg(OAc)_2_, 15% glycerol, and divided in two (samples 1 and 2). Sample 1 was adjusted to 10 mM EDTA 0.8% DBC, while sample 2 was adjusted with 0.8% DBC. After 15min on ice, the samples were centrifuged in the TL120.1 rotor (Beckman Coulter) at 100,000 rpm for 30 min at 4°C to pellet insoluble material and ribosomes/subunits. The supernatant from Sample 1 was saved as the “total ER protein” fraction (550 μl). The supernatant from sample 2, which has now been depleted of ∼80% of Sec61 via its ribosome association, was passed sequentially over two gravity flow columns containing 200 μl of protein A resin containing anti-Sec61β antibody pre-equilibrated in extraction buffer. The resulting flow through was collected and saved as the “Sec61-depleted ER protein” fraction (550 μl).

#### Proteoliposome Reconstitutions

Reconstitutions of proteoliposomes (or matched empty liposomes) were performed with minor modifications of previous methods (Görlich and Rapoport, 1993, Guna et al., 2018) as follows. Purified lipids were obtained from Avanti Polar Lipids and a 20 mg/ml stock suspension was prepared in 50 mM HEPES, pH 7.5, 15% glycerol, and 10 mM DTT containing Phosphatidyl-choline (PC; from bovine liver), Phosphatidyl-ethanolamine (PE; from bovine liver), and synthetic 1,2-dioleoyl-sn-glycero-3-phosphoethanolamine-N-lissamine rhodamine B (rhPE) in a 8:1.9:0.1 ratio. BioBeads-SM2 (BioRad) were prepared by first wetting them with methanol, then washing extensively with distilled water. After all traces of methanol were removed, the beads were adjusted with water so that the settled beads occupied 50% volume. For use in reconstitutions, the BioBeads were dispensed from this 50% slurry in the desired amount, and the excess liquid was removed by aspiration just before use. The volumes of BioBeads referred to below indicate the packed volume of beads.

For reconstitutions with total and Sec61-depleted ER proteins, the detergent-solubilized preparations from above were supplemented with 850 μg lipids from the prepared 20 mg/ml stock prepared as above. Control liposome reconstitutions contained extraction buffer instead of protein extracts. These mixtures were then added to ∼350 μl packed BioBeads (prepared as above) and incubated at 4°C for 18 h with gentle end-over-end mixing. The liquid was separated from the BioBeads, diluted with 4 volumes of ice-cold water, and centrifuged for 45 min at 75,000 rpm in a TL100.3 rotor (Beckman). The pellet was resuspended in 90 μl 100 mM KOAc, 50 mM HEPES pH 7.4, 1 mM Mg(OAc)_2_, 250 mM sucrose. The rhodamine-labeled PC was used to ensure equal membrane recovery, and protein content was visualized by SDS-PAGE followed by Sypro Ruby staining.

For reconstitutions with purified proteins, purified EMC (or its matched buffer control), purified SR (or its matched buffer control), DBC, and lipids were mixed in a final volume of 90 μl; the final mixture contained 0.52% DBC, 42 mM HEPES, pH 7.4, 333 mM KOAc, 44 mM NaCl, 2.67 mM Mg(OAc)2 and ∼2 pmol EMC and ∼1.5 pmol SR. This was added to 50 ul of BioBeads (packed volume) and incubated with gentle mixing for 16 h at 4°C. The liquid was separated from the BioBeads, diluted with 10 volumes of ice-cold water, and centrifuged for 45 min at 100,000 rpm in a TL100.3 rotor (Beckman). The pellet was resuspended in 15 μl 100 mM KOAc, 50 mM HEPES pH 7.4, 1 mM Mg(OAc)_2_, 250 mM sucrose, 1 mM DTT. The rhodamine-labeled PC was used to ensure equal membrane recovery, and protein content was visualized by SDS-PAGE and Sypro Ruby staining. The PLs were used immediately for functional assays without freezing.

#### Sequence analysis

All GPCRs and tail-anchored membrane proteins were retrieved from the curated and reviewed human Uniprot dataset (UniProt Consortium, 2018). GPCRs containing a signal sequence and tail-anchored proteins destined for mitochondria were manually removed from this set. This left 728 GPCRs and 235 tail-anchored proteins. The TMD regions were taken to be those annotated by Uniprot’s automated algorithms. Based on these designations, the length of the translocated domain and the charge within the flanking domains were determined. Relative hydrophobicity was determined using the transmembrane tendency method (Zhao and London, 2006). The charge difference was calculated using the difference between the C- and N-terminal flanking charges (Hartmann et al., 1989).

### Quantification and Statistical Analysis

Quantification of autoradiographs were performed on phosphorimager data using the gel analysis and lane plotting plugins of ImageJ. Percent translocation in Figure 2D was calculated by dividing the amount of the translocated product by the sum of the translocated and non-translocated products within each lane. Percent glycosylation in Figures 4B, S3E, and S4A was calculated by dividing the intensity of the glycosylated product by the sum of the glycosylated and unglycosylated products. EMC dependence (Figures 4A, 4E, and S5A) is defined as 100 x [1 – (%glycosylation in ΔEMC hRM)/(%glycosylation in wild-type hRM)]. Error bars in Figure 4A reflect the standard deviation of three independent measurements.

## Supplemental Information

Table S1. Sequences of TMD Regions Analyzed in This Study, Related to Figure 4 The GPCR-TMD1 cassette depicted in Figure 3A was populated with the first TMD and adjacent flanking regions (∼10 residues on either side) of the indicated GPCRs. The TMD regions of the model proteins bacterial leader peptidase (LepB) and ASGR1 are shown for comparison. The TMD region (as annotated by Uniprot) is underlined, and flanking basic and acidic residues are shown in red and blue, respectively. The calcuated TM tendency score and charge difference are indicated for each TMD region. Note that the Uniprot annotated TMD may not be precise in defining the boundaries, so parameters such as TMD length and hydrophobicity should be interpreted with this caveat in mind.

Table S2. Sequences of TMD Mutants Analyzed in This Study, Related to Figure 4 The β1AR-TMD1 and LepB constructs were mutated as indicated (green residues indicate changes). The calcuated TM tendency score and charge difference are indicated for each TMD region. The TMD is underlined. Note that the assignment of the TMD for β1AR is different from that indicated in Uniprot (Table S1) and is based on the known structure of β1AR. Although not shown here, we have verified that the effect of 3L and Δ3 are due to the increase in hydrophobicity and decrease in TMD length, respectively, and not to the specific residues that are mutated. This was done by mutating or deleting three other residues in the TMD to achieve the same approximate hydrophobicity and length.
