# Supplementary material for: EMC Is Required to Initiate Accurate Membrane Protein Topogenesis
Source: Cell. 2018 Nov 29;175(6):1507–1519.e16. doi: 10.1016/j.cell.2018.10.009 (PMC6269167; doi:10.1016/j.cell.2018.10.009)
Supplement: Table S1. Sequences of TMD Regions Analyzed in This Study, Related to Figure 4 — The GPCR-TMD1 cassette depicted in Figure 3A was populated with the first TMD and adjacent flanking regions (∼10 residues on either side) of the indicated GPCRs. The TMD regions of the model proteins bacterial leader peptidase (LepB) and ASGR1 are shown for comparison. The TMD region (as annotated by Uniprot) is underlined, and flanking basic and acidic residues are shown in red and blue, respectively. The calcuated TM tendency score and charge difference are indicated for each TMD region. Note that the Uniprot annotated TMD may not be precise in defining the boundaries, so parameters such as TMD length and hydrophobicity should be interpreted with this caveat in mind. [file mmc1.pdf]

Cell, Volume *175*

Supplemental Information

EMC Is Required to Initiate Accurate Membrane Protein Topogenesis

Patrick J. Chitwood, Szymon Juszkiewicz, Alina Guna, Sichen Shao, and Ramanujan S. Hegde

name	sequence	hydro. (TM tend.)	charge Δ(C-N)
LepB	ANMFALILVIATLVTGILWSVDKFFFAPKRRERQAAAQ	21.9	+3
ASGR1	PPPPQPMLQRLSSGPRLLLLSLGLSLLLLVVVSVIGSQNSQLQEELR	23.5	-3
V1BR	ATTPWMGRDEELAKVEIGVLATVLVLATGGNLAVLLTLGQLGRKRSRM	17.1	+5
VN1R5	LKLVIIENMAEIMLFSLDLLLFSTDILSFNFPSKMIKLPG	13.7	0
MTR1L	GSIGSKLPQPEYPPALIIFMFSAMVITIVVDLIGNSMVILAVTKNKKL	17.7	+3
OR3A2	AEFILLGLVQTEEMQPVVFVLLLFAYLVTTGGNLSILAAVLVEPKLHA	21.2	+4
OR2L5	ILLGLFPPSKIGLFLFILFVLIFLMALIGNLSMILLIFLDTHLHTPMY	34.6	-2
ACM1	PQITVLAPGKGPWQVAFIGITTGLLSLATVTGNLLVLISFKVNTELKT	18.0	0
O52N2	GFFILNGVPGLEATHIWISLPFSFMYIIAVVGNSGLISLISHEEALHR	17.6	0
β_1AR	RQVSAELLSQQWEAGMSLLMALVVLLIVAGNVLVIAAIGSTQRLQTLT	21.1	+1
ACTHR	QNTARQNSDSPRVVMPEEIFFTISIVGVLENLIVLLAVFKNKNLQAPM	17.3	+1
ADRB1	PASESPEPLSQQWTAGMGLLMALIVLLIVAGNVLVIVAIAKTPRLQTL	28.0	0
CNR2	SNPMKDYMILSGPQKTAVAVLCTLLGLLSALENVAVLYLILSSHQLRR	20.0	+1
O56A3	NSFVRSPSWQHWLSLPLSLLFLLAVGANTTLLMTIWLEASLHQPLYYL	17.2	-1
NK1R	TSEPNQFVQPAWQMVLWAAAYTVIVVTSVVGNVVVMWIILAHKRMRTV	20.5	+4
CCKAR	SLDQPRPSKEWQPAMQILLYSLIFLLSVLGNTLVITVLIRNKRMRTVT	25.8	+4
TAAR5	VQGSSPRTVHTLGIQLVIYLASAAGMLIIVLGNVFVAFAVSYFKALHT	21.7	0
ADA1A	SQSTQPPAPVQMSKAILLGVILGGLILFGVLGNILVILSVASHRHLHS	27.2	0
AGTR2	STLQSSQKPSDKHMDAIPILYYIIFVIGFLVNIVVVTLFSSQKGPKKV	27.3	+3

Table S1 - Sequences of TMD regions analyzed in this study (related to Fig. 4). The GPCR-TMD1 cassette depicted in Fig. 3A was populated with the first TMD and adjacent flanking regions (~10 residues on either side) of the indicated GPCRs. The TMD regions of the model proteins bacterial leader peptidase (LepB) and ASGR1 are shown for comparison. The TMD region (as annotated by Uniprot) is underlined, and flanking basic and acidic residues are shown in red and blue, respectively. The calcuated TM tendency score and charge difference are indicated for each TMD region. Note that the Uniprot annotated TMD may not be precise in defining the boundaries, so parameters such as TMD length and hydrophobicity should be interpreted with this caveat in mind.
