# Supplementary material for: EMC Is Required to Initiate Accurate Membrane Protein Topogenesis
Source: Cell. 2018 Nov 29;175(6):1507–1519.e16. doi: 10.1016/j.cell.2018.10.009 (PMC6269167; doi:10.1016/j.cell.2018.10.009)
Supplement: Table S2. Sequences of TMD Mutants Analyzed in This Study, Related to Figure 4 — The β1AR-TMD1 and LepB constructs were mutated as indicated (green residues indicate changes). The calcuated TM tendency score and charge difference are indicated for each TMD region. The TMD is underlined. Note that the assignment of the TMD for β1AR is different from that indicated in Uniprot (Table S1) and is based on the known structure of β1AR. Although not shown here, we have verified that the effect of 3L and Δ3 are due to the increase in hydrophobicity and decrease in TMD length, respectively, and not to the specific residues that are mutated. This was done by mutating or deleting three other residues in the TMD to achieve the same approximate hydrophobicity and length. [file mmc2.pdf]

name	sequence	hydro. (TM tend.)	charge Δ(C-N)
β_1AR	QVSAELLSQQWEAGMSLLMALVVLLIVAGNVLVIAAIGSTQRLQTLT	26.3	+3
1A	QVSAELLSQQWEAGMSLLMAAVVLLIVAGNVLVIAAIGSTQRLQTLT	24.9	+3
2A	QVSAELLSQQWEAGMSLLMAAVVALIVAGNVLVIAAIGSTQRLQTLT	23.4	+3
1L	QVSAELLSQQWEAGMSLLMALVVLLIVLGNVLVIAAIGSTQRLQTLT	27.7	+3
2L	QVSAELLSQQWEAGMSLLMALVVLLIVLLNVLVIAAIGSTQRLQTLT	29.8	+3
3L	QVSAELLSQQWEAGMSLLMALVVLLIVLLLVLVIAAIGSTQRLQTLT	33.2	+3
0	QVSAQLLSQQWQAGMSLLMALVVLLIVAGNVLVIAAIGSTQQLQTLT	26.3	0
+1	QVSAQLLSQQWQAGMSLLMALVVLLIVAGNVLVIAAIGSTQRLQTLT	26.3	+1
+2	QVSAELLSQQWQAGMSLLMALVVLLIVAGNVLVIAAIGSTQRLQTLT	26.3	+2
+4	QVSAELLSQQWEAGMSLLMALVVLLIVAGNVLVIAAIGRTQRLQTLT	26.3	+4
+5	QVSAELLSQQWEAGMSLLMALVVLLIVAGNVLVIAAIGRTQRLKTLT	26.3	+5
+6	QVSAELLSEQWEAGMSLLMALVVLLIVAGNVLVIAAIGRTQRLKTLT	26.3	+6
Δ1	QVSAELLSQQWEAGMSLLMALVVLLIVAG-VLVIAAIGSTQRLQTLT	27.9	+3
Δ2	QVSAELLSQQWEAGMSLLMALVVLLIVA--VLVIAAIGSTQRLQTLT	28.1	+3
Δ3	QVSAELLSQQWEAGMSLLMALVVLLIV---VLVIAAIGSTQRLQTLT	27.7	+3
LepB	ANMFALILVIATLVTGILWSVDKFFFAPKRRERQAAAQ	21.9	+3
LepB-Ext	ANMFALSILVGIATLVTGILTWSVDKFFFAPKRRERQAAAQ	20.8	+3

Table S2 - Sequences of TMD mutants analyzed in this study (related to Fig. 4). The β_1AR-TMD1 and LepB constructs were mutated as indicated (green residues indicate changes). The calcuated TM tendency score and charge difference are indicated for each TMD region. The TMD is underlined. Note that the assignment of the TMD for β_1AR is different from that indicated in Uniprot (Table S1) and is based on the known structure of β_1AR. Although not shown here, we have verified that the effect of 3L and Δ3 are due to the increase in hydrophobicity and decrease in TMD length, respectively, and not to the specific residues that are mutated. This was done by mutating or deleting three other residues in the TMD to achieve the same approximate hydrophobicity and length.
